# Palliative Care in Patients with Liver Cirrhosis: A Scoping Review of Identification, Interventions, and Implementation Barriers and Facilitators

**DOI:** 10.3390/healthcare14172761

**Published:** 2026-09-01

**Authors:** Birgitte Gade Jacobsen, Mai-Britt Guldin, Mette Munk Lauridsen, Lea Ladegaard Grønkjær

**Affiliations:** 1 Department of Regional Health Research, University of Southern Denmark, 6700 Esbjerg, Denmark; mette.enok.munk.lauridsen@rsyd.dk (M.M.L.); lea.ladegaard.gronkjaer@rsyd.dk (L.L.G.); 2 Department of Gastroenterology, University Hospital of Southern Denmark, 6700 Esbjerg, Denmark; 3 Department of Public Health, Aarhus University, 8000 Aarhus, Denmark; m.guldin@ph.au.dk

**Keywords:** liver cirrhosis, palliative care, identification, interventions, facilitators, barriers, scoping review

## Abstract

**Highlights:**

**What are the main findings?**

- Identification of palliative care needs is supported by structured tools and clinical criteria, but implementation remains inconsistent and palliative care is often introduced late due to prognostic uncertainty, fragmented care pathways, and limited palliative care competencies.
- Palliative care interventions across inpatient, outpatient, and home-based settings are associated with improved symptom management, communication, care planning, and care coordination.

**What are the implications of the main findings?**

- Integration of palliative care may improve symptom management, communication, and preparedness for patients with cirrhosis and their informal caregivers.
- Education, interdisciplinary collaboration, and integrated care pathways may support timely and consistent implementation of palliative care in hepatology services.

**Abstract:**

Background/objectives: Liver cirrhosis is a life-limiting, non-malignant condition with a high symptom burden, psychosocial challenges, and unpredictable disease trajectory. As such, patients with cirrhosis and their caregivers might benefit from palliative care (PC) interventions. However, in most clinical settings, PC is not yet an integral part of basic hepatology care. This scoping review aimed to map how patients with cirrhosis are identified for palliative care, which interventions are delivered, and which factors influence implementation. Methods: The review was conducted in accordance with Joanna Briggs Institute methodology and reported following the PRISMA-ScR guidelines. PubMed, CINAHL, and Scopus were searched. The initial search was conducted in November 2024 and updated in December 2025, June and August 2026. Quantitative, qualitative, and mixed-method studies involving adults (≥18 years) with liver cirrhosis, informal caregivers, and healthcare professionals were included. Data were charted descriptively and organized according to identification approaches, interventions, barriers, and facilitators. Results: In total, 39 studies were included. Patients were identified using identification tools, screening tools, clinical criteria, and prognostic scores. Interventions across inpatient, outpatient, and home-based settings included symptom management, prognostic communication, advance care planning, goals-of-care discussions, psychosocial support, and multidisciplinary collaboration. Overall, studies reported improvements in symptom management, communication, healthcare utilization, and informal caregiver outcomes. However, palliative care integration remained inconsistent and often occurred late. Key barriers included prognostic uncertainty, fragmented care pathways, limited training, and misconceptions about palliative care. Facilitators included education, structured assessment tools, and integration of palliative care into hepatology services. Conclusions: This review identified diverse approaches to patient identification and palliative care delivery, alongside key barriers and facilitators to implementation. Earlier integration of palliative care based on care needs, supported by communication, education, and interdisciplinary collaboration, may improve care for patients with cirrhosis.

## 1. Introduction

Liver disease represents a major and growing global health challenge. Worldwide, cirrhosis is among the leading causes of death and was responsible for approximately 1.5 million deaths in 2019, with an increasing trend observed in recent years [1]. The underlying causes of cirrhosis vary geographically with alcohol-associated liver disease (ALD), metabolic dysfunction-associated steatotic liver disease (MASLD), and chronic viral hepatitis representing major etiologies [2,3].

Cirrhosis is the final stage of chronic liver disease and progresses from a compensated phase with few or no symptoms to a decompensated stage marked by severe complications such as ascites, hepatic encephalopathy, infections, renal impairment, and variceal bleeding [4]. Patients experience a high symptom burden, cognitive and functional decline, reduced health-related quality of life (HRQoL) [5,6], and frequent unplanned hospitalizations [7,8,9,10]. Many patients report feelings of stigma, inadequate support from healthcare professionals, unmet needs and fear of what the future holds [11,12], while informal caregivers often experience significant emotional and physical burden [13,14,15,16,17].

Current management strategies for cirrhosis primarily focus on treating complications, alleviating symptoms, and evaluating eligibility for liver transplantation, which remains the only curative option for a limited proportion of patients [18,19].

Palliative care, as defined by the World Health Organization (WHO), aims to improve quality of life for patients and their informal caregivers facing life-limiting illness through early identification, comprehensive assessment, and management of physical, psychosocial, and spiritual needs [20]. Patients with cirrhosis face a high symptom burden, unpredictable disease progression, and complex social needs, often relying on informal caregivers. This stresses the appropriateness of early integration of palliative care alongside standard treatment [21,22]. Despite this clear clinical need, integration of palliative care into routine cirrhosis care remains inconsistent, with low referral rates and palliative care often introduced late in the disease trajectory, frequently near the end of life [23,24,25,26]. This delay is partly explained by challenges faced by hepatologists in identifying the optimal timing for palliative care in an often unpredictable disease course. In addition, limited understanding of the role and scope of palliative care among healthcare professionals, patients, and informal caregivers may further hinder its timely integration [24,25,26].

Previous reviews have addressed selected aspects of palliative care in patients with cirrhosis or end-stage liver disease (ESLD), including supportive and palliative care needs, patient, informal caregiver and healthcare professional perspectives, models of care, and palliative care interventions [27,28,29,30,31,32]. However, these reviews have focused on specific aspects of palliative care or provided narrative overviews. A broader synthesis integrating how patients are identified for palliative care, the range of interventions delivered across healthcare settings, and the barriers and facilitators influencing implementation is lacking. Such a synthesis may provide a more comprehensive understanding of how identification of palliative care needs relates to subsequent care delivery and of the factors that may hinder the translation of recognized needs into timely palliative care in routine hepatology practice.

Therefore, the objective of this scoping review is to map the existing evidence on palliative care for patients with cirrhosis. In line with scoping review methodology, the review focuses on the available evidence rather than intervention effectiveness or methodological quality assessment [33]. Specifically, the review aims to identify and describe how patients are assessed for palliative care, map the range of palliative care interventions delivered across settings, and explore reported facilitators and barriers to implementation from the perspectives of patients, informal caregivers, and healthcare professionals.

### Research Question

Q1: How are patients with cirrhosis identified for palliative care, and what interventions are provided?

Q2: What are the key facilitators and barriers to implementing palliative care interventions for patients with cirrhosis from the perspectives of patients, informal caregivers, and healthcare professionals? 

## 2. Materials and Methods

This scoping review was conducted in accordance with the Joanna Briggs Institute (JBI) methodology [33] and is reported in accordance with the Preferred Reporting Items for Systematic Reviews and Meta-Analyses Extension for Scoping Reviews (PRISMA-ScR) guidelines [34]. Following completion of the review, the manuscript was retrospectively registered in the Open Science Framework OSF (DOI 10.17605/OSF.IO/CSM5W) in July 2026. In line with JBI guidance, a protocol was developed and finalized prior to title/abstract screening to guide the review process but was not prospectively registered or published. The protocol specified the review objective, eligibility criteria, search strategy, study selection process, and data charting procedures. No major methodological deviations occurred during the review, apart from the planned literature search updates. A completed PRISMA-ScR checklist is provided as Supplementary Material.

### 2.1. Eligibility Criteria

The approach for developing eligibility criteria was inspired by Participants, Concept and Context (PCC) recommended for scoping reviews to define the target population, the phenomenon of interest, and the relevant clinical context [34]. Studies were eligible for inclusion if they involved adults (≥18 years) with liver cirrhosis and identified palliative care needs, irrespective of etiology and disease severity. The review was limited to adults, as palliative care in paediatric populations differs substantially from adult palliative care with respect to patient population, disease characteristics, and models of care. No restrictions on etiology or disease severity were applied to ensure that approaches to patient identification and palliative care delivery could be mapped across the full spectrum of cirrhosis and throughout the disease trajectory. No restrictions were applied regarding gender or ethnicity. Quantitative, qualitative, and mixed-method studies were included to capture different aspects of how palliative care eligibility is assessed and delivered to patients with cirrhosis. This included how palliative care needs are identified, the types of palliative care interventions provided, as well as facilitators and barriers to implementation from the perspectives of patients, informal caregivers, and healthcare professionals. Only peer-reviewed full-text articles published in English from 2014 onwards were include. The 2014 date restriction was applied to capture contemporary evidence reflecting current approaches to palliative care, including the increasing emphasis on earlier, needs-based integration alongside disease-directed treatment. Earlier studies may therefore reflect different conceptualizations and models of palliative care that are less representative of current practice.

### 2.2. Search Strategy

A systematic literature search was conducted in PubMed (National Library of Medicine), CINAHL (Ebsco), and Scopus (Elsevier) to identify peer-reviewed studies published in English from 2014 onwards. The eligibility criteria and literature search characteristics are summarized in Table 1. The search strategy combined controlled vocabulary (including MeSH terms where applicable) and free-text keywords related to cirrhosis and palliative care. Database-specific search strategies are provided in the Supplementary Material (Table S1). In PubMed, the database-specific ‘Adult: 19+ years’ filter was applied to restrict the search to adult populations. Although the review eligibility criterion was ≥18 years, the PubMed filter defines adults as individuals aged 19 years and older. Reference lists of included studies were also screened to identify additional relevant publications. The search strategy was reviewed by an experienced medical librarian. The initial search was conducted in November 2024 and updated in December 2025, June and August 2026.

### 2.3. Article Screening

Following the search, the records were imported into a reference management program (EndNote 21, Philadelphia, PA, USA: Clarivate Analytics; 2023) and duplicates were removed. The eligibility criteria were pilot tested by the first and last authors on the first 50 records to ensure a common understanding and consistent application of the inclusion and exclusion criteria. Following the pilot phase, titles and abstracts were screened independently by the first and last authors using the predefined eligibility criteria. Full-text articles of potentially relevant studies were subsequently retrieved and independently assessed for eligibility by the first and last authors. Throughout both title/abstract and full-text screening, uncertainties or disagreements regarding study eligibility were discussed between the two authors and resolved through consensus.

### 2.4. Data Charting

Data charting was conducted using a standardized data charting form developed by the first and last authors (Table S2). The form was piloted on a subset of included studies and refined iteratively during the charting process. These data included details on the authors and country, year of publication, study design, number of participants, patient and disease characteristics, description of the intervention, outcomes of significance to the review aim, and authors’ conclusions. Data charting was undertaken by the first author and subsequently reviewed by the last author. Any uncertainties or discrepancies identified during the review of the charted data were discussed between the first and last authors and resolved through consensus. Consistent with JBI guidance for scoping reviews, no critical appraisal of individual sources of evidence was performed [34].

### 2.5. Synthesis of Results

To address the research questions, the charted data were analyzed using complementary approaches. For studies examining how palliative care eligibility is assessed and interventions are delivered (RQ1), data were summarized descriptively to map key study characteristics, assessment approaches, and intervention types across the literature. In this review, “intervention” refers to any structured clinical, organizational, or supportive action intended to identify, address, or manage palliative care needs in patients with cirrhosis. For studies exploring facilitators and barriers to implementing palliative care (RQ2), an inductive thematic approach was used to synthesize findings from included studies. For the thematic synthesis, findings related to barriers and facilitators to palliative care implementation were extracted from the Results/Findings sections of the included studies and subsequently coded by the first author. Codes were organized according to perspectives from patients, informal caregivers, or healthcare professionals and grouped into broader categories and themes through an iterative process of comparison across studies. The preliminary coding and thematic structure were reviewed by the last author, and any uncertainties regarding coding or theme allocation were discussed until consensus was reached.

## 3. Results

**Figure 1:**
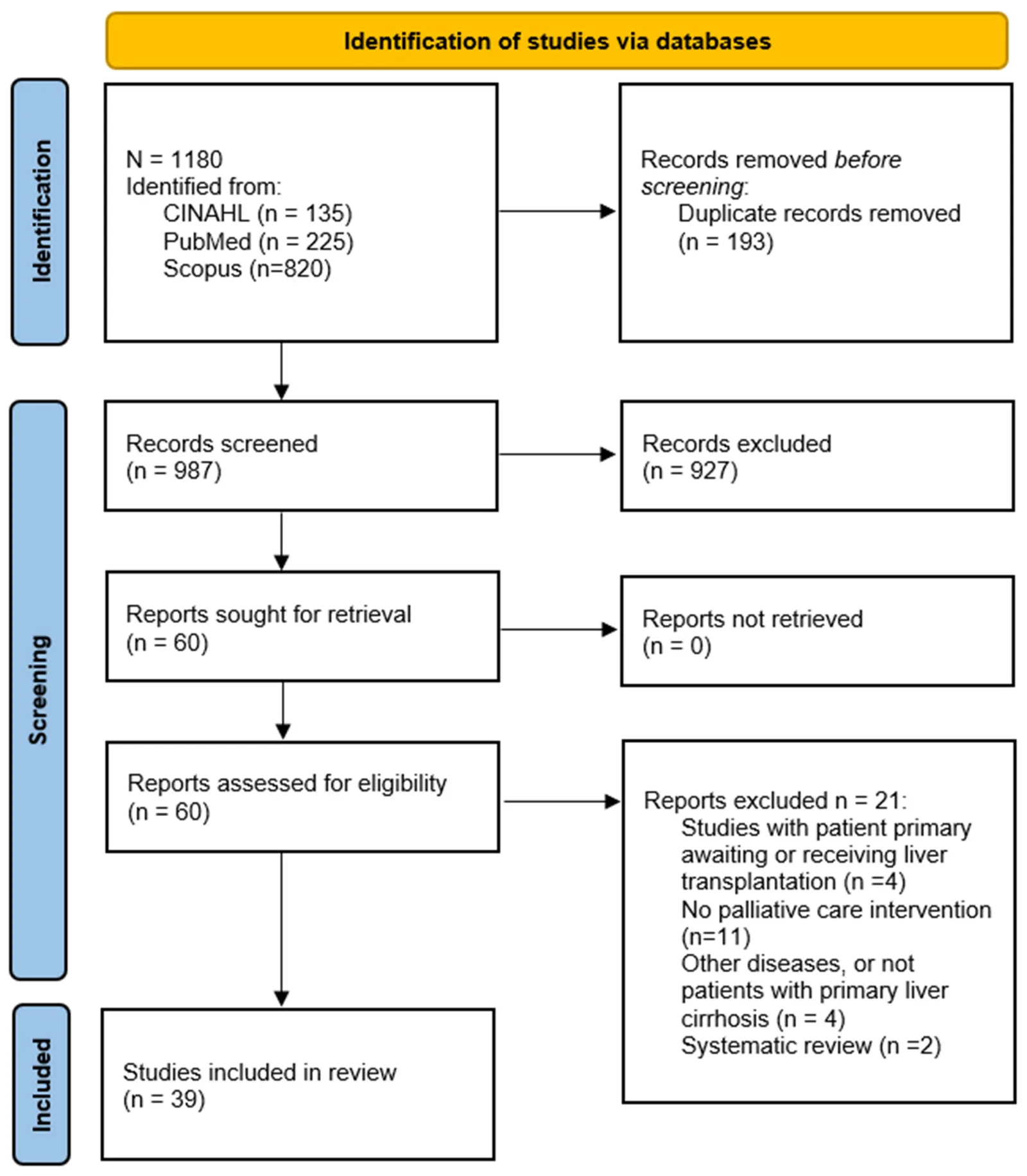
PRISMA 2020 flow diagram for study selection adapted from Page et al. [74].

A total of 1180 articles were identified through database searching. After eliminating duplicates, 987 articles were reviewed based on titles and abstracts, and 60 were identified for full-text assessment. After full-text reviews, 39 articles met the inclusion criteria. The flow diagram in Figure 1 summarizes the process.

The results are presented according to the two research questions. A total of 25 studies addressed assessment methods and palliative care interventions [35,36,37,38,39,40,41,42,43,44,45,46,47,48,49,50,51,52,53,54,55,56,57,58,59]. RQ1 is summarized descriptively, highlighting patterns in study characteristics, patient identification approaches, and types of interventions delivered. A total of 16 studies explored barriers and facilitators to implementation [43,52,60,61,62,63,64,65,66,67,68,69,70,71,72,73]. RQ2 is presented thematically, illustrating experiences, perceptions, and contextual factors influencing palliative care delivery. Two studies contributed to both research questions [43,52]. In line with the aim of a scoping review, the results describe and map the range of assessment approaches, intervention types, and implementation factors reported in the literature, rather than evaluating effectiveness.

### 3.1. Characteristics of Included Articles

A total of 174.490 participants were included across the 39 studies. The study populations comprised patients with cirrhosis (*n* = 172.279), informal caregivers (*n* = 222), and healthcare professionals (*n* = 1992).

Gender distribution was variably reported, with most studies including predominantly male patient populations. The mean age of patients ranged from approximately 47 to 73 years (mean age 60.0). Healthcare professionals included hepatologists, gastroenterologists, transplant surgeons, nurses, palliative care specialists, general practitioners, general internal medicine clinicians, and multidisciplinary team members with varying levels of experience.

Cirrhosis etiology was reported in most studies and was predominantly ALD, followed by MASLD and viral hepatitis (HBV/HCV). Less common etiologies include autoimmune liver disease and cryptogenic cirrhosis. Disease severity was reported in several studies, with mean Model of End-Stage Liver Disease (MELD) scores ranging from 11.62 to 25.2.

The included studies were conducted across inpatient, outpatient, community, and home-based settings, with some incorporating primary care perspectives. Studies originated from the United States, the United Kingdom, Australia, Portugal, and Canada. Study designs included qualitative interview studies, retrospective and prospective cohort studies, randomized controlled trials, mixed-methods studies, and quality improvement projects.

### 3.2. RQ1 Identification of Palliative Care Needs in Patients with Liver Cirrhosis

#### 3.2.1. Structured Screening and Assessment Tools

Across the included studies, structured screening and assessment tools were consistently used to support earlier and more systematic identification of palliative care needs. However, their ability to translate identification into palliative care referral varied across settings.

Use of Supportive and Palliative Care Indicators Tool (SPICT) with direct referral was associated with increased specialist palliative care involvement and community referrals, whereas use of SPICT followed by multidisciplinary discussion resulted in fewer planned interventions [41]. In a feasibility study, the SPICT supported structured patient identification, but healthcare professionals reported uncertainty about when to initiate palliative care conversations due to the unpredictable course of cirrhosis [55]. A retrospective cohort study applying modified SPICT criteria found that 62% of patients met criteria for palliative care need, but only 43% were referred to specialist palliative care services [53]. Another study suggested that SPICT-based referral criteria may be more useful for identifying patients with palliative care needs than traditional prognostic scores such as Child-Pugh and MELD [40] while MELD-Na alone was found to be insufficient for needs-based assessment in a third study [44]. Taken together, these findings suggest that structured identification tools may support more systematic identification of patients with palliative care needs than prognostic scores alone, although referral to palliative care remained inconsistent across studies.

The Surprise Question (SQ) (“Would you be surprised if this patient were to die in the next 12 months?”) was used to identify patients eligible for the study intervention [38,51]. Patients with a “No” response had lower 12-month survival, with nearly half dying within a year [38]. The SQ demonstrated high sensitivity and negative predictive value but low specificity and moderate positive predictive value. SQ responses generally reflected illness severity, as higher MELD scores were more frequently associated with “No” answers, supporting its role in identifying patients at higher risk [38]. However, across studies, the SQ was primarily used as a screening tool to support prognostic assessment and the identification of patients who may benefit from palliative care, rather than as a standalone decision-making tool.

The screening tool Necesidades Paliativas/Palliative Needs (NECPAL), which combines the SQ with clinical, functional, and psychosocial indicators, was used alongside the Chronic Liver Disease Questionnaire (CLDQ) to identify unmet palliative care needs and symptoms [37]. This approach was feasible for routine nursing assessments and supported identification of symptom burden, although systematic documentation of subsequent palliative actions remained limited.

Overall, the reviewed screening and assessment approaches indicate a shift from prognosis-based scores or identification towards multidimensional, needs-based assessment. While tools such as SPICT, the SQ, and NECPAL supported structured identification of patients with palliative care needs, their implementation did not consistently translate into timely palliative care referral or subsequent clinical action.

#### 3.2.2. Clinical Indicators and Prognostic Tools

Across the included studies, standardized clinical criteria were used to identify patients with cirrhosis eligible for palliative care. Two or more hospital admissions due to complications of cirrhosis within a 6-month period were used as an indicator of advanced disease, particularly in patients who are not eligible for liver transplantation [35,43]. In another study, eligibility for a collaborative care intervention was based on hospitalization with decompensated cirrhosis combined with poor quality of life (SF-36 < 40) [54]. A prognostic screening tool based on five clinical criteria (Child-Pugh C, ≥2 admissions, ongoing alcohol use, transplant ineligibility, WHO performance status 3–4) was applied for hospitalized patients with cirrhosis to identify risk of death within 12 months. A cumulative score of 3 or above was applied as the trigger for the intervention [39]. Another study used a modified version based on the same criteria. Results from the two studies showed that use of the tool supported identification of appropriate patients and was associated with increased palliative care specialist consultations and hospice referrals [39,42]. In contrast, the Bristol Prognostic Score, developed from the original five-item criteria [39], demonstrated lower predictive accuracy, underscoring that mortality prediction alone is insufficient for identifying palliative care needs [36].

Across studies, indicators and prognostic criteria were primarily used to identify patients at increased risk of clinical deterioration or death, whereas multidimensional assessment tools were more closely aligned with identifying unmet palliative care needs beyond prognosis alone.

#### 3.2.3. Symptoms, Quality of Life and Burden Assessment

Across studies, Patient-reported outcome measures and data (PRO/PROM data) were used to assess symptoms, psychological distress, health-related quality of life, prognostic communication and informal caregiver burden. Tools included PRO Palliative Care questionnaire (PRO-Pall), Edmonton Symptom Assessment System—revised (ESAS-r), Hospital Anxiety and Depression Scale (HADS), Short Form—Liver Disease Quality of Life (SF-LDQOL), McGill Quality of Life Questionnaire—Expanded/B version (MQOL-B) and EuroQol 5 Dimensions (EQ-5D), the Prognosis and Treatment Perception Questionnaire (PTPQ), General anxiety disorder (GAD-7), Patient Health Questionnaire (PHQ-9), Palliative care Outcome Scale—Carer version (POS Carer), Caregiver Quality of Life (CQOL) and Zarit Burden Interview (ZBI) [43,44,54,55,57,62]. Unlike prognostic tools, PRO/PROM measures primarily assessed quality of life, symptom burden, psychological distress, and supportive care needs, thereby complementing clinical assessment rather than predicting disease progression.

In a single study, PRO data on anxiety, depression, and quality of life remained stable during 6 month follow-up, but caregiver burden decreased markedly [55]. Similarly, another study found no significant overall improvements in quality of life, although per-protocol analyses showed improvements in physical functioning and fatigue at 3 months [54]. In other studies, PRO data revealed high baseline symptom burden, substantial psychological distress, and variable quality of life. Interventions were associated with reductions in pain and fatigue, improvements in anxiety, depression, and quality of life, and reduced caregiver burden together with improved caregiver-reported outcomes [43,44,57,62]. In a single study, the PTPQ specifically highlighted gaps in prognostic communication, with many patients reporting infrequent discussions about disease trajectory and end-of-life preferences, despite valuing these conversations [62].

Overall, the findings suggest that PRO/PROM measures and data contribute important information beyond prognosis by capturing multidimensional aspects of patient and caregiver experience, thereby supporting more comprehensive identification of palliative care needs.

### 3.3. RQ1 Palliative Care Interventions Delivered

#### 3.3.1. Outpatient Palliative Care Interventions

Outpatient interventions for patients with cirrhosis typically combined needs-based palliative assessment, symptom management, Advance Care Planning (ACP) and Goals of Care (GOC) discussions. A hepatologist-led ACP intervention targeting patients with decompensated cirrhosis without an existing advance directive (AD) reported an increase in AD completion from 8% to 31% and documented GOC discussions from 0% to 51% [56]. Studies integrating palliative care within hepatology services, through multidisciplinary clinics or hepatology-embedded programs, reported higher ACP completion, more documented GOC discussions, enhanced psychosocial assessments, increased hospice referrals, better medication coordination, and fewer hospital deaths [40,50]. A mixed-methods evaluation of an integrated outpatient hepatology–palliative care clinic reported reduced emergency department visits and hospitalizations following consultation, and 62.5% of deceased patients died outside the hospital. The intervention included symptom management, GOC discussions, future planning, and care coordination [52]. Another newly published large cluster randomized trial compared palliative care delivered by trained hepatologists with specialist palliative care and reported comparable changes in quality of life, symptom burden, and depression across both models. Patient satisfaction improved more in the hepatologist-led group, suggesting that hepatologist-delivered palliative care may be a feasible model for patients with ESLD [59]. Early outpatient referral and the use of decision aids, such as ACP video tools, were associated with increased patient knowledge and differences in preferences regarding life-sustaining treatments [35,49]. A pilot randomized trial evaluated a post-discharge collaborative care model supported by an interdisciplinary team, involving multidimensional assessment, individualized care plans, and regular follow-up for six months. Improvements in physical functioning, energy/fatigue, and physical component scores were reported in per-protocol analyses. No significant differences in quality of life, readmissions, or mortality between groups were observed between groups [54]. An outpatient model also reported findings suggesting possible benefits for informal caregivers, including greater perceived support, communication, care coordination, and preparedness for future care [52].

Overall, outpatient interventions shared several core components, including symptom management, ACP, GOC discussions, and care coordination. Although the organisation of these interventions differed, most models were integrated within existing hepatology services or delivered in close collaboration with hepatology teams.

#### 3.3.2. Inpatient Palliative Care Interventions

Hospitalized patients received palliative consultations as part of their inpatient care, addressing symptom management, ACP, GOC discussions, illness understanding, and optional social or spiritual support. Across two studies [42,48], these consultations were associated with increased palliative care utilization and hospice referrals, as well as decreased hospital admissions. However, individual study findings also showed that consultations were often infrequent and late, particularly for patients with higher disease severity [46]. Early inpatient palliative care programs that included post-discharge nurse follow-up reported changes in outcomes such as time to first readmission and days alive outside the hospital, despite challenges with recruitment and incomplete implementation [51].

Compared with outpatient models, inpatient interventions were generally initiated later in the disease trajectory and were more closely linked to acute hospitalization, although similar findings regarding care planning were reported.

#### 3.3.3. Home-Based Palliative Care Intervention

In a study by Oliveira et al., home-based palliative care was delivered through an integrated service, with patients receiving ongoing follow-up and monitoring at home by a specialized palliative care team. Patients receiving home-based palliative care were typically older and had more advanced disease, including hepatocellular carcinoma. The intervention was associated with significantly fewer emergency episodes and patients receiving home-based palliative care were more likely to die at home [45]. Although evidence was limited, home-based models appeared particularly relevant for patients with advanced disease and emphasized continuity of care beyond the hospital setting.

#### 3.3.4. Timing and Implementation

Across studies, palliative care for patients with cirrhosis was characterized by late and inconsistent integration into routine practice. In a large Veterans Health Administration cohort, GCD documentation increased over time, although only 29.8% had a documented GCD and the median time from first GCD to death was 37 days [58]. Similar patterns of late or incomplete integration were observed across identification approaches and care settings, suggesting that implementation challenges extended beyond the availability of assessment tools to their translation into routine clinical practice. Chart-based assessments indicated that recommended palliative care elements, including documentation of treatment preferences, ACP, and timely referral to specialist services, were only partially implemented, and many patients approached the final months of life without structured palliative involvement [47]. When palliative care was involved, studies reported more consistent documentation of GOC discussions, including code-status changes and assignment of healthcare proxy, as well as increased use of symptom-focused interventions. Reductions in hospital admissions were also reported following palliative referral [48].

Taken together, the findings indicate that structured identification tools and integrated palliative care models are increasingly available across hepatology settings. However, their implementation remains inconsistent, with variation in referral practices, intervention delivery, and timing of palliative care integration.

An overview of findings from RQ1 is listed in Table 2.

### 3.4. RQ2 Perspectives of Patients and Informal Caregivers

#### 3.4.1. Barriers

- Knowledge, attitudes, and perceptions

Across several studies [60,61,64], patients and informal caregivers demonstrated limited knowledge of palliative care, often associating it specifically with hospice or end-of-life care. These studies also reported limited understanding of disease progression and ACP processes, and some patients expressed concerns that referral to palliative care might signal a loss of active treatment or that ACP represented “giving up” [48,49,52].

Informal caregivers in individual studies described restricted access to information about disease progression, inadequate symptom management [64,65], and insufficient support after the patient’s death [64].

Across multiple studies [60,63,64,68], patients reported unmet emotional, informational, and psychosocial needs, including inadequate symptom management and limited understanding of disease progression. Existing healthcare services were described as poorly aligned with patients’ palliative and supportive care needs, and healthcare professionals were reported to have limited prior experience with specialized palliative care [60,63,64,68].

Collectively, these findings indicate that patients’ and informal caregivers’ unmet needs were influenced not only by symptom burden but also by limited knowledge of palliative care, inadequate communication regarding disease progression and future care, and healthcare services that were insufficiently responsive to supportive and palliative care needs.

- Timing of palliative care discussions

Across multiple studies, infrequent end-of-life discussions and inadequate communication were linked to worse PRO, including higher symptom burden, anxiety, depression, and lower quality of life [57,61]. Evidence from two studies showed that ACP and Goals of Care Designation (GCD) discussions during acute admissions were often poorly recalled, with hepatic encephalopathy identified as a barrier. Patients preferred these discussions to occur early and during stable periods in the disease trajectory, particularly in outpatient settings [60,68]. Discussing end-of-life issues with informal caregivers was also described as challenging, and preferences for information and involvement in ACP varied among patients [60]. Across studies, patients consistently preferred palliative care discussions to occur earlier and during clinically stable phases rather than during acute hospital admissions, highlighting timing as an important determinant of patient engagement.

#### 3.4.2. Facilitators

- Education

Patient and informal caregivers believed palliative care and ACP could improve preparation and support [61,64], and Donlan et al. suggested possible benefits of palliative care education for patients’ and informal caregivers’ understanding and acceptance of early integration alongside active treatment [61].

- Nurse-led supportive care

Kimbell et al. found that a nurse-led supportive care intervention, combining regular follow-up, symptom and psychosocial support, care coordination, and anticipatory care planning, may support patients’ and informal caregivers’ coping and understanding of palliative care, while facilitating future care planning and reducing uncertainty [43].

- Communication, information and coordinated care

Communication style and frequency matters. Realistic, empathic and direct information about prognosis and future care needs, informal caregiver involvement, and coordinated care were reported essential and preferred among most patients in several studies [60,63,64,68]. Frequent and high-quality prognostic communication between hepatologists, patients, and informal caregivers was associated with higher quality of life among patients, lower caregiver burden and anxiety and supported treatment decisions, preparation for the future, maintaining hope, and coping with illness [57]. Similarly, integrated palliative care models were perceived positively by patients and informal caregivers related to collaboration and communication [52]. In one study, patients and informal caregivers reported improved communication, collaboration, care coordination, symptom management, future planning, and emotional support as key facilitators of implementation [52].

Overall, facilitators identified by patients and informal caregivers consistently centered on communication, education, and coordinated care rather than on specific intervention models, suggesting that how palliative care is delivered may be as important as which intervention is provided.

### 3.5. RQ2 Perspectives of Healthcare Professionals

#### 3.5.1. Barriers

- Unpredictable disease trajectory, low confidence, and lack of training

In a single study, healthcare professionals, including hepatologists, palliative care specialists, and general practitioners, reported that referrals to specialist palliative care were often infrequent and late [70]. Contributing factors included unpredictable disease trajectory that made it difficult to identify the appropriate timing for palliative care discussions and referral.

Across two studies, healthcare professionals described low confidence in discussing prognosis and palliative care, as well as limited knowledge and formal training, which contributed to communication barriers and uncertainty about how to initiate conversations about prognosis and future care [67,71]. A multidisciplinary survey similarly identified specialty-based differences in confidence in symptom management and assessment of goals, as well as differences in perceived ownership of PC delivery [73]. Although most healthcare professionals supported palliative care, several studies identified barriers including patient perceptions that associated palliative care with end-of-life care, as well as fear, anxiety, and limited clinical guidance [67,70,71]. Fragmented care, poor continuity, and unclear care pathways were also reported as barriers to implementation [71].

Overall, healthcare professionals described organizational and professional barriers rather than a lack of willingness to provide palliative care. Across studies, uncertainty about prognosis, fragmented care pathways, and limited palliative care competencies appeared to delay timely integration despite general support for palliative care.

- Role of general practitioners in palliative care

One study highlighted that general practitioners were willing to take a larger role in end-of-life care, as they recognized this patient population as particularly vulnerable due to a substantial symptom burden, social isolation, and stigma factors that exacerbate caregiver strain and constrain available end-of-life care options. However, their involvement remained limited because care continued to be dominated by specialists. The general practitioners emphasized the need for clearer prognostic communication, defined care pathways, and better access to specialist support [69]. In another study, family physicians described a reactive approach to cirrhosis care, shaped by fragmented care pathways and uncertainty about when to introduce palliative care discussions. They emphasized the need for clearer prognostic communication, practical tools, defined care pathways, and better access to specialist support [72]. These findings indicate that primary care professionals perceived a greater potential role in palliative care than current care pathways allowed, emphasizing the importance of clearer responsibilities and collaboration across healthcare sectors.

#### 3.5.2. Facilitators

- Nurse-led supportive intervention

Kimbell et al. found that a nurse-led supportive care intervention was reported to support coordination between services, supported timely decision-making, and was perceived by healthcare professionals as feasible and acceptable in routine practice [43].

- Relevant training and education across sectors

Palliative care-trained hepatologists reported that they felt better equipped to provide primary palliative care and that successful integration requires time, cross-disciplinary education, and strong collaborations [66]. Hepatologists, palliative care specialists, and general practitioners reported distinct training needs, including communication and symptom management, to improve confidence and support implementation of palliative care [67]. Palliative care specialists and general practitioners required greater understanding of the unpredictable trajectory of cirrhosis and the effects of impaired liver function [69,71,72].

- Integration of care

Stronger collaboration between hepatology, primary care, and palliative care services, together with integrated care models, was consistently identified as facilitating implementation through improved communication, care coordination, and future planning across patients, informal caregivers, and healthcare professionals [52,55,67,69].

Across studies, interdisciplinary collaboration, education, and integration of palliative care within existing hepatology pathways were commonly identified as factors supporting implementation.

An overview of findings from RQ2 is listed in Table 3.

### 3.6. Summary of Findings

The 39 included studies varied in study design, patient populations, and care contexts, reflecting the complexity of integrating palliative care into hepatology service. Overall, palliative care was inconsistently integrated into routine practice, with substantial variation in assessment approaches, timing of referral, and the scope of interventions offered. Across studies, palliative care interventions were associated with reported improvements in symptom management and communication about prognosis, ACP and GOC discussions, as well as increased hospice referrals. Palliative care interventions for patients with cirrhosis showed a consistent tendency toward improved symptom management, enhanced communication about prognosis, ACP and GOC discussions, and increased hospice referrals across settings. Despite these reported findings, the included studies indicated a pattern of late and inconsistent introduction of palliative care, with some patients receiving limited structured support. PRO data demonstrated high symptom burden and psychological distress at baseline, while studies incorporating these assessments reported improvements in symptoms, mental well-being, and quality of life following palliative care interventions. Some studies also reported reductions in caregiver burden and improvements in caregiver-reported outcomes.

Patients and informal caregivers demonstrated limited knowledge of palliative care in several studies, frequently associating it with end-of-life care, and reported unmet emotional, informational, and psychosocial needs linked to delayed or insufficient communication about prognosis and future care. Clear communication and education were associated with better understanding, preparation, and acceptance of palliative care.

Healthcare professionals described the unpredictable disease trajectory, limited training, low confidence in palliative discussions, and fragmented care pathways as key barriers. At the same time, cross-disciplinary collaboration, improved coordination, and enhanced palliative care training were identified as important facilitators of earlier and more consistent integration.

## 4. Discussion

Despite the availability of several structured identification tools and palliative care intervention models, implementation remained inconsistent across the included studies. By integrating evidence on patient identification, interventions, and implementation barriers and facilitators, this review highlights a gap between identifying palliative care needs and translating these needs into routine clinical practice. This suggests that the challenge lies not only in identifying patients and defining appropriate interventions, but also in the organisational, professional, and conceptual conditions required for their timely integration.

### 4.1. Identification of Palliative Care Needs and Interventions Delivered (RQ1)

Identification of palliative care needs in chronic non-malignant diseases is a well-described challenge, as palliative care is often introduced late due to the absence of clear needs-based criteria [75]. This challenge is particularly evident in cirrhosis, where the unpredictable and fluctuating disease trajectory complicates timely identification. Consistent with previous research, prognostic uncertainty emerged as a major barrier to early integration [24,25].

The findings of this review highlight the complementary roles of prognostic and needs-based approaches to patient identification. Prognostic tools and the SQ were useful for identifying patients at increased risk of deterioration. However, evidence from both the included studies and studies outside this review indicates that the SQ is most appropriately used as a screening tool rather than a standalone prognostic or referral tool because of its limited prognostic accuracy when used in isolation [38,76]. Taken together, these findings reinforce previous evidence that, given the prognostic uncertainty inherent to cirrhosis, prognosis alone is insufficient to identify patients with unmet supportive and palliative care needs [24,25].

In contrast, multidimensional tools such as SPICT and NECPAL extend assessment beyond prognosis by incorporating broader patient needs [40,41,53]. This is consistent with recommendations of early palliative care, where referral is guided by patient needs rather than prognosis alone [17,20].

However, an important finding of this review was that structured identification did not consistently translate into palliative care referral or implementation. For example, fewer than half of patients meeting modified SPICT criteria were referred to specialist palliative care services [53]. These findings suggest that improving identification tools alone is unlikely to substantially increase palliative care integration unless accompanied by clear referral pathways, organisational support, and confidence among healthcare professionals to initiate palliative care.

In addition, findings of this review suggest that PRO measures complement clinical assessment by systematically capturing symptom burden, psychological distress, and broader supportive care needs [39,40,49]. Despite these benefits, PRO data was not systematically incorporated across studies, suggesting that symptom assessment remains underutilized as part of routine palliative care integration.

Across care settings, the included studies described a broad range of palliative care interventions, most commonly combining symptom management, psychosocial support, ACP, GOC, GCD and care coordination. Although several interventions reported potential benefits in patient- and caregiver-related outcomes, intervention models varied considerably with respect to timing, organisation, and healthcare professionals involved, making comparisons across studies difficult [46,47,48,56,71]. This heterogeneity is consistent with previous systematic reviews’ findings and recent international recommendations, which highlight uncertainty regarding the optimal model of palliative care delivery while advocating early, needs-based integration into hepatology care [17,22,28]. The variation across intervention models may therefore reflect not only uncertainty about the optimal model of care, but also differences in how palliative care is organised and integrated within existing hepatology services. This suggests that successful implementation may depend less on a single intervention model and more on whether palliative care responsibilities, referral pathways, and interdisciplinary collaboration are clearly embedded within routine care.

A recently published cluster randomized trial reported comparable outcomes in quality of life, symptom burden, and depression between hepatologist-delivered and specialist-delivered palliative care [59]. These findings support current recommendations that hepatologists should be equipped to provide primary palliative care, with specialist palliative care reserved for patients with more complex needs [17]. Similarly, multidisciplinary and nurse-led models were generally perceived as feasible, supporting recommendations that palliative care should be delivered through multidisciplinary collaboration integrated within routine hepatology care, while also illustrating the practical challenges of implementing and evaluating palliative care interventions in patients with advanced cirrhosis [54]. However, evaluating such models in advanced cirrhosis remains challenging, as disease progression, mortality, and transplantation may complicate recruitment, retention, and follow-up [54].

In addition, several studies reported reduced caregiver burden and improved preparedness among informal caregivers, highlighting the importance of addressing caregiver needs alongside those of patients [43,52,55,57]. This is in line with current recommendations and evidence emphasising that support for informal caregivers should be considered an integral component of palliative care for patients with advanced liver disease [17,20,22,64]. Taken together, these findings suggest that while multiple approaches for identifying palliative care needs and delivering palliative interventions are increasingly available, integration of palliative care into routine hepatology practice remains variable and has not yet been systematically implemented.

### 4.2. Barriers and Facilitators Influencing Implementation (RQ2)

Implementation of palliative care in cirrhosis was consistently reported as variable across studies, reflecting a persistent gap between identification of needs and their translation into routine clinical practice.

- Conceptual and terminological barriers

A key barrier across studies was the widespread perception of palliative care as synonymous with end-of-life care among patients, informal caregivers, and healthcare professionals [48,49,52,55,62,63,73]. Previous studies have shown that when framed as “supportive care”, interventions are perceived more positively and are associated with a greater willingness to engage compared to when described as “palliative care” [68,69]. This suggests that terminology may influence how palliative care is perceived and accepted, potentially affecting its timely integration.

Terminological inconsistency has also been identified as a barrier in previous research, particularly regarding the use of terms such as “palliative care”, “supportive care”, and “end-of-life care” [77]. This variation was also observed within the included studies, where palliative care was used alongside or interchangeably with “supportive care” [39,40,43,47,64,65]. In addition, related concepts such as ACP, GOC, GCD, anticipatory care planning, end-of-life discussions, personal directives and prognostic communication were also used inconsistently across studies [35,39,40,42,43,46,47,48,49,50,51,57,58,60,62,63,64,68,69,72]. Such conceptual inconsistency may create uncertainty about what palliative care encompasses and when it should be introduced, potentially complicating its integration into routine hepatology care.

Among patients and informal caregivers, these perceptions of palliative care were associated with fear and reluctance to engage, while healthcare professionals described uncertainty regarding referral and challenges initiating conversations about prognosis and future care planning. Similar barriers have been identified in previous research, including limited understanding and negative perceptions of palliative care, as well as challenges among healthcare professionals in communicating about prognosis and future care [24,27,70]. These findings are also consistent with WHO guidance highlighting misconceptions about palliative care and cultural and social perceptions of death and dying as barriers to its integration [20]. Together, these findings suggest that conceptual understandings of palliative care may influence both patient acceptance and professional practice.

Definitions of “early” palliative care also varied considerably across studies [35,38,39,44,48,51,60,62,63,67,68,71], ranging from prognostic-based identification of patients at risk of death within one year [32,34,35,36] to definitions based on specialist involvement within 30 days before death [35]. In a condition characterized by unpredictable trajectories, such prognostic-based definitions may not align with needs-based approaches and may reinforce late referral patterns [78].

When palliative care is primarily understood as end-of-life care, its potential role earlier in the disease trajectory may be overlooked, helping to explain why integration often occurs late despite available tools and interventions. Although the term “supportive care” may reduce negative connotations, terminology alone may not overcome the broader professional and organisational barriers to early palliative care integration.

- Healthcare professionals and organizational barriers

Healthcare professionals reported uncertainty regarding timing and initiation of palliative care discussions, as well as limited confidence in communication [67,70,71], which may contribute to the late referrals observed across studies. This suggests that the challenge extends beyond identifying patients with palliative care needs to translating this identification into clinical practice. Organizational and professional barriers included fragmented care pathways, limited training, lack of clear referral processes, and resource limitations [52,66,67,70,71,72]. Similar barriers have been identified in previous systematic reviews, including limited professional confidence, communication challenges, unclear professional roles, and fragmented care coordination [27,28]. Low et al. [27] further highlighted the need for clearer interdisciplinary pathways and better coordination between hepatology, palliative care, and primary care. Current international recommendations similarly emphasise palliative care training, multidisciplinary collaboration, and integration of palliative care within hepatology services [17,22]. Taken together, these findings suggest that identification of palliative care needs alone is insufficient without organisational mechanisms that enable identified needs to result in appropriate clinical action.

- Facilitators of integration

In contrast, the included studies suggest that integration of palliative care in cirrhosis is both feasible and acceptable when supported by improved coordination, interdisciplinary collaboration, and palliative care training. These findings indicate that several of the professional and organisational barriers identified above may be modifiable. Education of healthcare professionals is associated with increased use of hospice services, suggesting that competency development influences clinical decision-making and referral patterns [79], while targeted training for hepatologists has been associated with reported improvements in knowledge, self-efficacy, and palliative care integration [80]. In addition, patients, caregivers, and healthcare professionals reported high satisfaction with an integrated hepatology–palliative care clinic model and highlighted benefits related to communication, collaboration, and care coordination [52]. This highlights the potential value of embedding palliative care competencies and responsibilities within existing hepatology services rather than relying solely on referral to specialist palliative care.

Overall, the findings suggest that successful integration of early palliative care in cirrhosis depends not only on identifying patients with unmet needs, but also on addressing conceptual barriers, strengthening professional competencies, and establishing organisational structures that support palliative care as part of routine hepatology practice.

### 4.3. Strengths and Limitations

This scoping review has several strengths. It includes 39 studies identified through a comprehensive literature search. As such, it provides a comprehensive overview of how palliative care is identified, delivered, and implemented in patients with liver cirrhosis across a broad range of study designs and healthcare settings. By including both qualitative and quantitative studies, the review captures not only measurable outcomes but also the experiences and perspectives of patients, informal caregivers, and healthcare professionals. This aligns with the methodological purpose of scoping reviews to map complex and heterogeneous fields.

The review was conducted using a systematic approach guided by established methodological frameworks for scoping reviews, including the Joanna Briggs Institute methodology [33,34]. This supports the methodological rigour of the review process. The inclusion of multiple databases further supports the comprehensiveness of the search strategy.

However, several limitations should be considered. The absence of a prospectively registered and publicly available protocol limits the external transparency and reproducibility of the review process, as readers cannot directly compare the planned methodology with the final review. Although the protocol was developed and finalized before screening, prospective registration would have provided greater transparency regarding the planned review process.

This review was limited to peer-reviewed full-text articles published in English from 2014 onwards. Consequently, relevant evidence from non-English publications, grey literature, service evaluations, and conference abstracts may have been omitted. As implementation evidence may also be reported outside the peer-reviewed literature, these restrictions may have limited the identification of interventions, and implementation barriers and facilitators. In addition, the PubMed ‘Adult: 19+ years’ filter did not fully correspond to the re-view eligibility criterion of ≥18 years and may therefore have resulted in the omission of some potentially relevant records involving 18-year-old participants. The extent of any resulting selection bias cannot be determined.

The heterogeneity of the included studies in terms of design, populations, and outcomes limited the ability to directly compare findings across studies. As a result, the synthesis is primarily descriptive, and no conclusions about effectiveness can be drawn. Most included studies were small, single-center observational or feasibility studies, limiting transferability and making it difficult to determine which intervention components may be most effective.

Variability in how palliative care and “early” integration were defined across studies may have influenced the interpretation of findings. In particular, some studies relied on prognostic criteria, while others applied needs-based approaches, making it challenging to establish a consistent understanding of timing and eligibility.

Not all studies reported detailed participant characteristics, such as cirrhosis etiology, disease severity, or demographic variables, which may limit the generalizability of the findings. In addition, most studies were conducted in high-income countries, reducing the applicability of the results to other healthcare contexts.

Finally, as with all scoping reviews, no formal critical appraisal of study quality was undertaken, in accordance with guidance from the Joanna Briggs Institute. Therefore, the findings should be interpreted as a mapping of the available evidence rather than an evaluation of its methodological quality.

### 4.4. Future Directions for Research and Implications for Clinical Practice

This review highlights that the main challenge is no longer whether palliative care is relevant for patients with cirrhosis, but how it can be integrated systematically and earlier in routine hepatology care. Future research should therefore move beyond describing needs and focus on developing, testing, and implementing models of integrated palliative care that are feasible in everyday clinical practice.

A key research priority is to establish consensus-based, needs-driven referral criteria that can be applied consistently across settings. Although several identification tools and clinical criteria were identified, evidence remains limited regarding their applicability across clinical settings and their role in supporting timely integration of palliative care and patient-centered care. Future studies should compare needs-based assessment strategies, including structured screening tools and PRO/PROM, and evaluate their impact on symptom burden, quality of life, healthcare utilisation, and caregiver outcomes.

In addition, there is a need for implementation research examining how palliative care can be embedded within hepatology services. Future studies should evaluate integrated care pathways, hepatology-led primary palliative care models, nurse-led interventions, and collaborative models involving hepatology, specialist palliative care, and primary care. Particular attention should be given to identifying which implementation strategies may improve uptake, sustainability, and equity of access across healthcare settings.

From a clinical perspective, the findings support a shift from prognosis-based referral practices towards routine needs-based assessment. Rather than waiting until end-of-life becomes imminent, palliative care principles should be incorporated alongside standard hepatology care when patients experience significant symptom burden, psychosocial concerns, recurrent decompensation, or increasing care needs. Structured identification tools and regular assessment of PRO/PROM may support earlier identification of patients who could benefit from palliative care.

The findings further suggest that palliative care should be regarded as a core component of cirrhosis management rather than an optional specialist service. To achieve this, hepatology services may benefit from implementing structured pathways for ACP, GOC, prognostic communication, and symptom assessment, supported by interdisciplinary collaboration and targeted education of healthcare professionals. Strengthening palliative care competencies among hepatologists, nurses, and primary care clinicians may reduce uncertainty, support communication with patients and caregivers, and facilitate more timely and consistent integration of palliative care throughout the disease trajectory.

## 5. Conclusions

This scoping review identified a range of tools and approaches used to identify palliative care needs in patients with cirrhosis, including structured screening tools, prognostic criteria, and PRO/PROM measures and data. In addition, a broad range of palliative care interventions delivered across outpatient, inpatient, and home-based settings was identified, although considerable variation existed in how these approaches were applied in clinical practice.

Implementation of palliative care in cirrhosis was influenced by multiple interacting barriers and facilitators. Key barriers included prognostic uncertainty, inconsistent terminology, limited palliative care competencies, fragmented care pathways, and perceptions of palliative care as end-of-life care among patients, informal caregivers, and healthcare professionals. Facilitators included interdisciplinary collaboration, structured care pathways, and education of healthcare professionals.

Overall, by integrating evidence on patient identification, palliative care interventions, and implementation barriers and facilitators, this review highlights a persistent gap between identifying palliative care needs and translating these needs into routine clinical practice. Given the heterogeneity of the available evidence, further evaluative research is needed to determine the effectiveness of palliative care interventions and how they can best be implemented within routine hepatology care.

## Figures and Tables

**Table 1:** Search Strategy.

Criteria	Inclusion
**Study design**	Peer-reviewed studies: qualitative, quantitative, and mixed-methods
**Language**	English
**Patient population**	Adults (≥18 years) with liver cirrhosis, palliative care need, any etiology or disease severity
**Phenomena of** **interest**	Assessment of palliative care eligibility and delivery. Palliative care interventions. Barriers and facilitators to implementation
**Databases**	PubMed (National Library of Medicine), CINAHL (Ebsco), Scopus (Elsevier)
**Publication period**	2014 onwards
**Search updates**	November 2024; December 2025; June and August 2026

**Table 2:** Identification approaches and palliative care interventions in cirrhosis.

Domain	Approach	Main Findings	Key Contributing Studies
**Identification**			
	SPICT, SQ, NECPAL	Structured screening and assessment tools supported systematic identification, but referral and subsequent palliative care actions remained inconsistent	[37,40,41,53,55]
	Clinical criteria	Use and predictive performance of clinical indicators and prognostic tools varied across studies	[36,39,42,43,54]
	PRO/PROMs.	Supported identification of symptom burden and psychosocial needs, with variable findings for quality of life and caregiver burden.	[43,44,55,62]
**Interventions**			
	ACP	ACP engagement and documentation varied across studies, while some interventions reported increased completion of AD and care planning documentation.	[35,40,42,43,46,47,48,49,50,51,55,56,60,63,64,68,72]
	GOC	GOC discussions were inconsistently implemented and documented, although some interventions reported increased GOC documentation and recording of treatment preferences.	[40,42,46,48,49,50,51,56,58,60,68,72]
	Prognostic communication	Gaps in prognostic communication were identified, while communication supported prognostic understanding, future planning, decision-making, and coping	[57,62]
	Symptom management	Reductions in symptom burden and improvements in quality of life were reported in some studies.	[35,37,44,46,47,48,50,51,59,64,72]
	Care coordination and integrated care models	Improved care coordination and fewer emergency visits or hospitalizations were reported in some studies.	[40,45,46,50,51,52]
**Timing**			
		Palliative care was often initiated late and inconsistently integrated into routine cirrhosis care.	[47,48,58]

**Table 3:** Barriers and facilitators influencing implementation of palliative care in cirrhosis.

Perspective	Key Barriers	Key Facilitators	Key Contributing Studies
**Patients and** **informal** **caregivers**			
	Limited knowledge and misconceptions about palliative care and ACP. Concerns that ACP implies “giving up”. Limited recall of ACP/GOC discussions during acute admissions	Education and information about palliative care. Nurse-led support. Early, realistic, and empathic communication, including timely ACP/GOC discussions. Family involvement. Coordinated care	[43,52,57,60,61,64,68]
**Healthcare** **professionals**			
	Late and infrequent referrals. Prognostic uncertainty. Limited confidence and training in palliative care and prognostic communication. Fragmented care pathways and unclear referral processes. Limited involvement of general practitioners	Palliative care education and training. Nurse-led intervention. Clear referral pathways. Interdisciplinary collaboration between hepatology, primary care, and palliative care specialist	[43,52,66,67,69,70,71,72,73]

## Data Availability

All charted data are available in the Supplementary Material (Table S1). The data were extracted from published studies included in the review.

## Supplementary Materials

The following supporting information can be downloaded at: https://www.mdpi.com/article/10.3390/healthcare14172761/s1, Table S1: Search Strategy. Table S2: Data charting from the included studies.

## Abbreviations

The following abbreviations are used in this manuscript:

ACP	Advance care planning
AD	Advance Directive
AIH	Autoimmune hepatitis
ALD	Alcohol-associated liver disease
BPS	Bristol Prognostic Score
CLDQ	Chronic Liver Disease Questionnaire
CQOL	Carer quality of life
EQ-5D-5L	EuroQol 5-dimension 5-level
ESAS-r	Edmonton Symptom Assessment System revised
ESLD	End-stage liver disease
GAD-7	General anxiety disorder
GCD	Goals of Care Designation
GOC	Goals Of Care discussions
HADS	Hospital Anxiety and Depression Scale
HCC	Hepatocellular carcinoma
Hep B/HBV	Hepatitis B virus
Hep C/HCV	Hepatitis C virus
HRQoL	Health-related quality of life
MASLD	Metabolic dysfunction-associated steatotic liver disease
MELD	Model for End-Stage Liver Disease
MQOL-B	McGill Quality of Life Questionnaire—Expanded/B version
NECPAL	Necesidades Paliativas/Palliative Needs
PHQ-9	Patient Health Questionnaire
PD	Personal directive
PRO-pall	PRO Palliative Care questionnaire
POS	Palliative care Outcome Scale
POS Carer	Palliative care Outcome Scale—Carer version
PTPQ	Prognosis and Treatment Perceptions Questionnaire
RNCM	Registered nurse case manager
SF-LDQOL	Short Form Liver Disease Quality of Life
SPICT	Supportive and Palliative Care Indicators Tool
SQ	Surprise Question
ZBI-12	Zarit Burden Interview-12

## References

[1] Liu Y.B., Chen M.K. (2022). Epidemiology of liver cirrhosis and associated complications: Current knowledge and future directions. World J. Gastroenterol..

[2] Moon A.M., Singal A.G., Tapper E.B. (2020). Contemporary Epidemiology of Chronic Liver Disease and Cirrhosis. Clin. Gastroenterol. Hepatol..

[3] Tan Y., Hu Y., Yang Y., Chu H. (2025). Alcohol-Related Liver Disease and Metabolic Dysfunction-Associated Steatotic Liver Disease: Molecular Pathogenesis and Therapeutic Interventions. MedComm.

[4] Ginès P., Krag A., Abraldes J.G., Solà E., Fabrellas N., Kamath P.S. (2021). Liver cirrhosis. Lancet.

[5] Younossi Z.M., Boparai N., Price L.L., Kiwi M.L., McCormick M., Guyatt G. (2001). Health-related quality of life in chronic liver disease: The impact of type and severity of disease. Am. J. Gastroenterol..

[6] Labenz C., Toenges G., Schattenberg J.M., Nagel M., Huber Y., Marquardt J.U., Galle P.R., Wörns M.A. (2019). Health-related quality of life in patients with compensated and decompensated liver cirrhosis. Eur. J. Intern Med..

[7] Wong E.L., Cheung A.W., Leung M.C., Yam C.H., Chan F.W., Wong F.Y., Yeoh E.K. (2011). Unplanned readmission rates, length of hospital stay, mortality, and medical costs of ten common medical conditions: A retrospective analysis of Hong Kong hospital data. BMC Health Serv. Res..

[8] Volk M.L., Tocco R.S., Bazick J., Rakoski M.O., Lok A.S. (2012). Hospital readmissions among patients with decompensated cirrhosis. Am. J. Gastroenterol..

[9] Di Pascoli M., Ceranto E., De Nardi P., Donato D., Gatta A., Angeli P., Pontisso P. (2017). Hospitalizations Due to Cirrhosis: Clinical Aspects in a Large Cohort of Italian Patients and Cost Analysis Report. Dig. Dis..

[10] Lovett G.C., Ha P., Roberts A.T., Bell S., Liew D., Pianko S., Sievert W., Le S.T.T. (2023). Healthcare utilisation and costing for decompensated chronic liver disease hospitalisations at a Victorian network. Intern Med. J..

[11] Grønkjær L.L., Lauridsen M.M. (2021). Quality of life and unmet needs in patients with chronic liver disease: A mixed-method systematic review. JHEP Rep..

[12] Valery P.C., Clark P.J., McPhail S.M., Rahman T., Hayward K., Martin J., Horsfall L., Volk M.L., Skoien R., Powell E. (2017). Exploratory study into the unmet supportive needs of people diagnosed with cirrhosis in Queensland, Australia. Intern. Med. J..

[13] Fabrellas N., Moreira R., Carol M., Cervera M., de Prada G., Perez M., Vazquez E., Sola M., Sancho R., Juanola A. (2020). Psychological Burden of Hepatic Encephalopathy on Patients and Caregivers. Clin. Transl. Gastroenterol..

[14] Hansen L., Chang M.F., Hiatt S., Dieckmann N.F., Lee C.S. (2024). Informal Family Care Partner Well-Being Is Diminished in End-Stage Liver Disease. Nurs. Res..

[15] Hansen L., Chang M., Hiatt S., Rosenkranz S., Dieckmann N., Lee C. (2025). Longitudinal changes in physical and psychological outcomes in spousal vs. non-spousal caregivers for patients with end-stage liver disease. Qual. Life Res..

[16] Volk M.L. (2020). Burden of Cirrhosis on Patients and Caregivers. Hepatol. Commun..

[17] Verma S., Pinter M., Macken L., Künzler-Heule P., Gluud L.L., Takkenberg B., Nunes R., Gheorghe L., Prentice W., Hudson B. (2026). EASL position paper on palliative care in advanced chronic liver disease. JHEP Rep..

[18] Ufere N.N., Halford J.L., Caldwell J., Jang M.Y., Bhatt S., Donlan J., Ho J., Jackson V., Chung R.T., El-Jawahri A. (2020). Health Care Utilization and End-of-Life Care Outcomes for Patients with Decompensated Cirrhosis Based on Transplant Candidacy. J. Pain Symptom Manag..

[19] D’Amico G., Garcia-Tsao G., Pagliaro L. (2006). Natural history and prognostic indicators of survival in cirrhosis: A systematic review of 118 studies. J. Hepatol..

[20] WHO Definition of Palliative Care. https://www.who.int/health-topics/palliative-care.

[21] Tandon P., Walling A., Patton H., Taddei T. (2021). AGA Clinical Practice Update on Palliative Care Management in Cirrhosis: Expert Review. Clin. Gastroenterol. Hepatol..

[22] Rogal S.S., Hansen L., Patel A., Ufere N.N., Verma M., Woodrell C.D., Kanwal F. (2022). AASLD Practice Guidance: Palliative care and symptom-based management in decompensated cirrhosis. Hepatology.

[23] Chen H., Johnston A., Palmer A., Mickenbecker M., O’Sullivan T., Clark P. (2021). Too little, too late: Palliation and end-stage liver disease. J. Gastroenterol. Hepatol..

[24] Rakoski M.O., Volk M.L. (2015). Palliative care for patients with end-stage liver disease: An overview. Clin. Liver Dis..

[25] Poonja Z., Brisebois A., Van Zanten S.V., Tandon P., Meeberg G., Karvellas C.J. (2014). Patients with Cirrhosis and Denied Liver Transplants Rarely Receive Adequate Palliative Care or Appropriate Management. Clin. Gastroenterol. Hepatol..

[26] Brown C., Khan S., Parekh T.M., Muir A.J., Sudore R.L. (2026). Barriers and Strategies to Effective Serious Illness Communication for Patients with End-Stage Liver Disease in the Intensive Care Setting. J. Intensive Care Med..

[27] Low J.T.S., Rohde G., Pittordou K., Candy B., Davis S., Marshall A., Stone P. (2018). Supportive and palliative care in people with cirrhosis: International systematic review of the perspective of patients, family members and health professionals. J. Hepatol..

[28] Haire E., Mann M., Yeoman A., Atkinson C., Wright M., Noble S. (2024). Supportive and palliative care needs in advanced non-malignant liver disease: Systematic review. BMJ Support. Palliat. Care.

[29] Mudumbi S.K., Bourgeois C.E., Hoppman N.A., Smith C.H., Verma M., Bakitas M.A., Brown C.J., Markland A.D. (2018). Palliative Care and Hospice Interventions in Decompensated Cirrhosis and Hepatocellular Carcinoma: A Rapid Review of Literature. J. Palliat. Med..

[30] Al-Moussally F., Tejada N., Khan S., Mandalia A., Kazi S. (2025). Palliative care in cirrhosis of the liver. BMJ Support. Palliat. Care.

[31] Sarria-Gómez D., Martínez Torres C.C., Estrada-Bermúdez D., Saavedra L. (2026). Early Palliative Care Integration in End-Stage Liver Disease: A Narrative Review of Clinical Strategies for Symptom Control and Quality of Life. J. Pain Palliat. Care Pharmacother..

[32] Sousa L., Silva S.M., Rego F., Nunes R., Oliveira H.M. (2025). Palliative Care in End-Stage Liver Disease. Livers.

[33] Peters M.D.J., Marnie C., Tricco A.C., Pollock D., Munn Z., Alexander L., McInerney P., Godfrey C.M., Khalil H. (2020). Updated methodological guidance for the conduct of scoping reviews. JBI Evid. Synth..

[34] Tricco A.C., Lillie E., Zarin W., O’Brien K.K., Colquhoun H., Levac D., Moher D., Peters M.D., Horsley T., Weeks L. (2018). PRISMA Extension for Scoping Reviews (PRISMA-ScR): Checklist and Explanation. Ann. Intern. Med..

[35] Barnes A., Woodman R.J., Kleinig P., Briffa M., To T., Wigg A.J. (2020). Hepatobiliary and Pancreatic: Early palliative care referral in patients with end-stage liver disease is associated with reduced resource utilization. J. Gastroenterol. Hepatol..

[36] Bowers S.P., Clare K., Hagerty L., McColl K., Smith E., Brown-Kerr A., Ahmed A., Finlay F., Dillon J.F., Barclay S. (2022). Predicting 1-year mortality among patients with decompensated cirrhosis: Results of a multicentre evaluation of the Bristol Prognostic Score. BMJ Open Gastroenterol..

[37] Gaines S., Satyshur R.D., Fitzpatrick S. (2025). Integration of Palliative Care Screening Tools for End-Stage Liver Patients to Improve End-of-Life Care. J. Dr. Nurs. Pract..

[38] Homann S., Pfaff J., Stovicek E., Agarwal R., Misra S.K., Pulley J.M., Siemann J.K., Spann A., Tillman S., Gatto C.L. (2025). Evaluating Performance of the Surprise Question to Predict 12-Month Mortality in Patients with End-Stage Liver Disease. Am. J. Hosp. Palliat. Med..

[39] Hudson B.E., Ameneshoa K., Gopfert A., Goddard R., Forbes K., Verne J., Collins P., Gordon F., Portal A.J., Reid C. (2017). Integration of palliative and supportive care in the management of advanced liver disease: Development and evaluation of a prognostic screening tool and supportive care intervention. Frontline Gastroenterol..

[40] Kearney A., Tiwari N., Cullen O., Legg A., Arbi I., Douglas C., Leggett B., Fenech M., Mina J., Hoey P. (2023). Improving palliative and supportive care in advanced cirrhosis: The HepatoCare model of integrated collaborative care. Intern. Med. J..

[41] Ward E., Hanbury G., Possamai L., Rassam T. (2025). Improving access to inpatient palliative care for patients with end-stage liver disease: A quality improvement project. BMJ Open Qual..

[42] Johnson A.W., Byriel B., Rubeck J., Ghabril M., Orman E.S. (2023). Standardized Criteria Increases Palliative Care Consultation Utilization in Patients with End-Stage Liver Disease: A Pilot Study. Am. J. Hosp. Palliat. Med..

[43] Kimbell B., Murray S.A., Byrne H., Baird A., Hayes P.C., MacGilchrist A., Finucane A., Brookes Young P., O’Carroll R.E., Weir C.J. (2018). Palliative care for people with advanced liver disease: A feasibility trial of a supportive care liver nurse specialist. Palliat. Med..

[44] Oliveira H.M., Ribeiro F., Lopes G., Frias E., Andrade F., Guiomar V., Eiras E., Rego M.F., Nunes R. (2025). Symptom burden in end-stage liver disease: A prospective cohort study of the symptoms experienced by patients and the role of palliative care. Ther. Adv. Gastroenterol..

[45] Oliveira H.M., Rocha C., Rego M.F., Nunes R. (2025). Palliative Homecare in Chronic Liver Disease: A Cohort Analysis of Factors and Outcomes Associated with Home Palliative Care in Patients with End-Stage Liver Disease. J. Palliat. Care.

[46] Orman E.S., Yousef A., Xu C., Shamseddeen H., Johnson A.W., Nephew L., Ghabril M., Desai A.P., Patidar K.R., Chalasani N. (2022). Palliative Care, Patient-Reported Measures, and Outcomes in Hospitalized Patients with Cirrhosis. J. Pain Symptom Manag..

[47] Patel A., Asch S., Antonio A.L., Kanwal F., Lorenz K., Riopelle D., Dickey A., Larkin J., Lee M., Walling A. (2020). Measuring the Quality of Palliative Care for Patients with End-Stage Liver Disease. Dig. Dis. Sci..

[48] Shehadah A., Yu Naing L., Bapaye J., Malik S., Mohamed M., Khalid N., Munoz A., Jadhav N., Mushtaq A., Okolo P. (2024). Early palliative care referral may improve end-of-life care in end-stage liver disease patients: A retrospective analysis from a non-transplant center. Am. J. Med. Sci..

[49] Ufere N.N., Robinson B., Donlan J., Indriolo T., Bloom J., Scherrer A., Mason N.M., Patel A., Lai J.C., Chung R.T. (2022). Pilot Randomized Controlled Trial of an Advance Care Planning Video Decision Tool for Patients with Advanced Liver Disease. Clin. Gastroenterol. Hepatol..

[50] Van Zyl C., Storms A.D., Van Deen W., Cardenas V., Ellis R., Flores A., Donovan J., Chu L., Patel T., Enguidanos S. (2023). A Pilot Study of a Palliative Care Service Embedded in a Hepatology Clinic at a Large Public Hospital. J. Palliat. Med..

[51] Shinall M.C., Karlekar M., Martin S., Gatto C.L., Misra S., Chung C.Y., Porayko M.K., Scanga A.E., Schneider N.J., Ely E.W. (2019). COMPASS: A Pilot Trial of an Early Palliative Care Intervention for Patients with End-Stage Liver Disease. J. Pain Symptom Manag..

[52] Warmels G., Wills A., Bruni A., Cohen L., Kelly E., Downar J., Patel A.A., Dargavel G., Rauthu S., Isenberg S.R. (2026). A mixed methods process evaluation of a collaborative outpatient palliative care clinic for patients with end-stage liver disease. BMC Palliat. Care.

[53] Reeves S.H., Ngu N., Le S., Runacres F. (2026). Palliative care in cirrhosis of the liver: An Australian perspective. BMJ Support. Palliat. Care.

[54] Orman E.S., Desai A.P., Kuchana S.K., Campbell N., Fowler N.R., McCarty J., Pike F., Mosesso K., Hotchkiss T., Chalasani N.P. (2026). The Cirrhosis Medical Home: A Pilot Randomized Trial of a Collaborative Care Model for Patients with Decompensated Cirrhosis. Clin. Transl. Gastroenterol..

[55] Grønkjær L.L., Jacobsen B.G., Teisner A.S., Bager P., Hamberg M.L., Mendahl J., Marsaa K., Guldin M.B., Kimer N., Lauridsen M.M. (2026). The LiverCare intervention: Implementing structured palliative care in hepatology—A multicenter mixed-methods feasibility study in patients with cirrhosis. BMC Palliat. Care.

[56] Patel A., Kogekar N., Agarwal R., Cohen C., Esteban J.P., Pourmand K., Tsai E., Harty A., Pelham-Braithwaite A., Perumalswami P. (2020). Improving Advance Care Planning in Outpatients with Decompensated Cirrhosis: A Pilot Study. J. Pain Symptom Manag..

[57] Li L., Donlan J., Nurhussien L., Horick N., Zeng C., Indriolo T., Armstrong M.E., Bizup G., Dalvi A., Zhu E. (2026). Prognostic communication, Psychological distress, and caregiving burden among caregivers of patients with decompensated cirrhosis. Liver Transplant..

[58] Patel A.A., O’Leary J.G., Bajaj J.S., Prause N., Hannum R., Noll A., Cheung R., Giustini A.B., Jakab S.S., Nobbe A. (2025). Increasing Goals of Care Discussions Among Veterans with Cirrhosis: A Veterans Health Administration Quality Improvement Initiative. Am. J. Gastroenterol..

[59] Verma M., Navarro V., Kosinski A., Taddei T., Kalman R., Barritt A.S., Jakab S., Serper M., Orman E., Balakrishnan M. (2026). Palliative Care Intervention for Patients with End-Stage Liver Disease: A Cluster Randomized Clinical Trial. JAMA Intern. Med..

[60] Carbonneau M., Davyduke T., Spiers J., Brisebois A., Ismond K., Tandon P. (2018). Patient Views on Advance Care Planning in Cirrhosis: A Qualitative Analysis. Can. J. Gastroenterol. Hepatol..

[61] Donlan J., Ufere N.N., Indriolo T., Jackson V., Chung R.T., El-Jawahri A., Traeger L. (2021). Patient and Caregiver Perspectives on Palliative Care in End-Stage Liver Disease. J. Palliat. Med..

[62] Donlan J., Kaplan A., Noll A., Pintro K., Horick N., Zeng C., Edelen M., Soetan Z., Comrie C., Indriolo T. (2025). Prognostic Communication, Symptom Burden, Psychological Distress, and Quality of Life Among Patients with Decompensated Cirrhosis. Clin. Gastroenterol. Hepatol..

[63] Malik S., Arutla V., Alamin T., Warraich F., Syed T.A., Nepal M., Ashraf M.F., Dunnigan K.J. (2025). Beyond the Diagnosis: A Deep Dive into the End Stage Liver Disease Experience from the Patient Perspective. Am. J. Hosp. Palliat. Med..

[64] Hudson B., Hunt V., Waylen A., McCune C.A., Verne J., Forbes K. (2018). The incompatibility of healthcare services and end-of-life needs in advanced liver disease: A qualitative interview study of patients and bereaved carers. Palliat. Med..

[65] Kimbell B., Boyd K., Kendall M., Iredale J., Murray S.A. (2015). Managing uncertainty in advanced liver disease: A qualitative, multiperspective, serial interview study. BMJ Open.

[66] Hoppmann N., Bakitas M., Stockdill M., DeNofrio J., Navarro V., Verma M. (2026). Palliative Care for Advanced Liver Disease: Hepatology and Palliative Care Specialists Experiences. J. Pain Symptom Manag..

[67] Low J., Vickerstaff V., Davis S., Bichard J., Greenslade L., Hopkins K., Marshall A., Thorburn D., Jones L. (2016). Palliative care for cirrhosis: A UK survey of health professionals’ perceptions, current practice and future needs. Frontline Gastroenterol..

[68] Sprange A., Ismond K.P., Hjartarson E., Chavda S., Carbonneau M., Kowalczewski J., Watanabe S.M., Brisebois A., Tandon P. (2020). Advance Care Planning Preferences and Readiness in Cirrhosis: A Prospective Assessment of Patient Perceptions and Knowledge. J. Palliat. Med..

[69] Standing H., Jarvis H., Orr J., Exley C., Hudson M., Kaner E., Hanratty B. (2017). How can primary care enhance end-of-life care for liver disease? Qualitative study of general practitioners’ perceptions and experiences. BMJ Open.

[70] Ufere N.N., Donlan J., Waldman L., Patel A., Dienstag J.L., Friedman L.S., Corey K.E., Hashemi N., Carolan P., Mullen A.C. (2019). Physicians’ Perspectives on Palliative Care for Patients with End-Stage Liver Disease: A National Survey Study. Liver Transplant..

[71] Low J., Davis S., Vickerstaff V., Greenslade L., Hopkins K., Langford A., Marshall A., Thorburn D., Jones L. (2017). Advanced chronic liver disease in the last year of life: A mixed methods study to understand how care in a specialist liver unit could be improved. BMJ Open.

[72] Barber T., Toon L., Tandon P., Green L.A. (2026). Family physicians’ integration of palliative principles in cirrhosis care: A cognitive task analysis study of symptom management. J. Can. Assoc. Gastroenterol..

[73] Saravanan N., Ganger D., Chen K., Orbuch R., Pirola A., Cheung A., Hauser J., Zhao L., Ladner D., Ufere N. (2026). Integrating Palliative Care in End-Stage Liver Disease: An Interprofessional Perspective on Communication, Terminology, and Educational Gaps. J. Palliat. Med..

[74] Page M.J., McKenzie J.E., Bossuyt P.M., Boutron I., Hoffmann T.C., Mulrow C.D., Shamseer L., Tetzlaff J.M., Akl E.A., Brennan S.E. (2021). The PRISMA 2020 statement: An updated guideline for reporting systematic reviews. BMJ.

[75] Karagkounis C., Connor S., Papadatou D., Bellali T. (2025). Evaluating Palliative Care Needs in Patients with Advanced Non-Malignant Chronic Conditions: An Umbrella Review of Needs Assessment Tools. Healthcare.

[76] Gupta A., Burgess R., Drozd M., Gierula J., Witte K., Straw S. (2024). The Surprise Question and clinician-predicted prognosis: Systematic review and meta-analysis. BMJ Support Palliat. Care.

[77] Robert R., Goldberg M. (2021). Palliative, palliative or palliative?. Crit. Care.

[78] Kircher C.E., Hanna T.P., Tranmer J., Goldie C.E., Ross-White A., Moulton E., Flegal J., Goldie C.L. (2025). Defining “early palliative care” for adults diagnosed with a life-limiting illness: A scoping review. BMC Palliat. Care.

[79] Chuang M.-H., Lee F.-N., Shiau Y.-T., Shen H.-Y., Lee C.-C., Chen S.S.-S., Huang S.-J. (2022). Physician Palliative Education Associated with High Use of Hospice Care Service. Am. J. Hosp. Palliat. Med..

[80] DeNofrio J.C., Verma M., Kosinski A.S., Navarro V., Taddei T.H., Volk M.L., Bakitas M., Ramchandran K. (2022). Palliative Care Always: Hepatology—Virtual Primary Palliative Care Training for Hepatologists. Hepatol. Commun..

