# Supplementary material for: Palliative Care in Patients with Liver Cirrhosis: A Scoping Review of Identification, Interventions, and Implementation Barriers and Facilitators
Source: Healthcare (Basel). 2026 Sep 1;14(17):2761. doi: 10.3390/healthcare14172761 (PMC13566022; doi:10.3390/healthcare14172761)
Supplement: Supplementary file 1 [file healthcare-14-02761-s001.zip › Tabel S1_Search strategy_proofread_BGJ.pdf]

Table S1 – Search Strategy

**Pubmed**<br>**Search 1: Initial search, conducted 28.11.2024**	
**Query**	**Results**
((((("Palliative Care"[Mesh]) OR ("palliati*"[Title/Abstract])) OR ("end of life care"[Title/Abstract])) OR ("advance care planning"[Title/Abstract])) OR (symptom burden[Title/Abstract])) OR (supportive care[Title/Abstract])	177,704
(("Liver Cirrhosis"[Mesh]) OR (hepatic cirrho*[Title/Abstract])) OR (end stage liver disease*[Title/Abstract])	125,289
((((("Palliative Care"[Mesh]) OR ("palliati*"[Title/Abstract])) OR ("end of life care"[Title/Abstract])) OR ("advance care planning"[Title/Abstract])) OR (symptom burden[Title/Abstract])) OR (supportive care[Title/Abstract])) AND ((("Liver Cirrhosis"[Mesh]) OR (hepatic cirrho*[Title/Abstract])) OR (end stage liver disease*[Title/Abstract]))	695
((((("Palliative Care"[Mesh]) OR ("palliati*"[Title/Abstract])) OR ("end of life care"[Title/Abstract])) OR ("advance care planning"[Title/Abstract])) OR (symptom burden[Title/Abstract])) OR (supportive care[Title/Abstract])) AND ((("Liver Cirrhosis"[Mesh]) OR (hepatic cirrho*[Title/Abstract])) OR (end stage liver disease*[Title/Abstract])) Filters: in the last 10 years, English, Humans, Adult: 19+ years	170
**Search 2: updated 10.12.2025**	
((((("Palliative Care"[Mesh]) OR ("palliati*"[Title/Abstract])) OR ("end of life care"[Title/Abstract])) OR ("advance care planning"[Title/Abstract])) OR (symptom burden[Title/Abstract])) OR (supportive care[Title/Abstract])) AND ((("Liver Cirrhosis"[Mesh]) OR (hepatic cirrho*[Title/Abstract])) OR (end stage liver disease*[Title/Abstract])) AND (2024/01/11:2025/10/12[pdat]) Filters: English, Humans, Adult: 19+ years, from 2024/01/11:2025/10/12	34
**Search 3: updated 15.06.2026**	
((((("Palliative Care"[Mesh]) OR ("palliati*"[Title/Abstract])) OR ("end of life care"[Title/Abstract])) OR ("advance care planning"[Title/Abstract])) OR (symptom burden[Title/Abstract])) OR (supportive care[Title/Abstract])) AND ((("Liver Cirrhosis"[Mesh]) OR (hepatic cirrho*[Title/Abstract])) OR (end stage liver disease*[Title/Abstract])) AND (2025/10/12:2026/15/06[pdat])Filters: English, Humans, Adult: 19+ years, from 2025/10/12:2026/15/06	16
**Search 4: updated 20.08.2026**	
((((("Palliative Care"[Mesh]) OR ("palliati*"[Title/Abstract])) OR ("end of life care"[Title/Abstract])) OR ("advance care planning"[Title/Abstract])) OR (symptom burden[Title/Abstract])) OR (supportive care[Title/Abstract])) AND ((("Liver Cirrhosis"[Mesh]) OR (hepatic cirrho*[Title/Abstract])) OR (end stage liver disease*[Title/Abstract])) AND (2025/10/12:2026/20/08[pdat])Filters: English, Humans, Adult: 19+ years, from 2026/15/06:2026/20/08	5

**Scopus**<br>**Search 1: Initial search, conducted 28.11.2024**	
**Query**	**Results**
TITLE-ABS-KEY (("liver cirrho*" OR "hepatic cirrho*" OR "end stage liver disease"))	81,866
TITLE-ABS-KEY ((palliati* OR "end of life care" OR "advance care planning" OR "supportive care" OR "symptom burden")))	111,687
TITLE-ABS-KEY ("liver cirrho*" OR "hepatic cirrho*" OR "end stage liver disease") AND TITLE-ABS-KEY ((palliati* OR "end of life care" OR "advance care planning" OR "supportive care" OR "symptom burden")))	1,131
TITLE-ABS-KEY (("liver cirrho*" OR "hepatic cirrho*" OR "end stage liver disease")) AND TITLE-ABS-KEY ((palliati* OR "end of life care" OR "advance care planning" OR "supportive care" OR "symptom burden"))) AND (LIMIT-TO (EXACTKEYWORD , "Humans") OR LIMIT-TO (EXACTKEYWORD , "Adult")) AND (LIMIT-TO (LANGUAGE , "English")) AND (LIMIT-TO (DOCTYPE , "ar")) AND PUBYEAR > 2013 AND PUBYEAR < 2025	643
**Search 2: updated 10.12.2025**	

Query	Results
TITLE-ABS-KEY (("liver cirrho*" OR "hepatic cirrho*" OR "end stage liver disease")) AND TITLE-ABS-KEY ((palliati* OR "end of life care" OR "advance care planning" OR "supportive care" OR "symptom burden"))) AND (LIMIT-TO (EXACTKEYWORD , "Humans") OR LIMIT-TO (EXACTKEYWORD , "Adult")) AND (LIMIT-TO (LANGUAGE , "English")) AND (LIMIT-TO (DOCTYPE , "ar")) AND PUBYEAR > 2024 AND PUBYEAR < 2026	96
**Search 3: updated 15.06.2026**	
**Query**	**Results**
TITLE-ABS-KEY (("liver cirrho*" OR "hepatic cirrho*" OR "end stage liver disease")) AND TITLE-ABS-KEY ((palliati* OR "end of life care" OR "advance care planning" OR "supportive care" OR "symptom burden"))) AND (LIMIT-TO (EXACTKEYWORD , "Humans") OR LIMIT-TO (EXACTKEYWORD , "Adult")) AND (LIMIT-TO (LANGUAGE , "English")) AND (LIMIT-TO (DOCTYPE , "ar")) AND PUBYEAR > 2025 AND PUBYEAR < 2027	53
**Search 4: updated 20.08.2026**	
**Query**	**Results**
TITLE-ABS-KEY (("liver cirrho*" OR "hepatic cirrho*" OR "end stage liver disease")) AND TITLE-ABS-KEY (((palliati* OR "end of life care" OR "advance care planning" OR "supportive care" OR "symptom burden")))) AND (LIMIT-TO (EXACTKEYWORD , "Humans") OR LIMIT-TO (EXACTKEYWORD , "Adult")) AND (LIMIT-TO (LANGUAGE , "English")) AND (LIMIT-TO (DOCTYPE , "ar")) AND PUBYEAR > 2025 AND PUBYEAR < 2027<br><br>Note: As Scopus allowed restriction by publication year rather than exact publication dates, the search covering 2026 was rerun in full on 20 August 2026. Records already identified in the previous search were excluded from the updated screening.	81

**Cinahl (EBSCOhost)**<br>**Search 1: Initial search, conducted 28.11.2024**	
**Query**	**Results**
**Query (expanded/display term):** (((((TX (MH "Liver Cirrhosis+")) OR (XB ("liver cirrhosis"))) OR (AB liver N3 cirrhosis)) OR (AB hepatic N3 cirrhosis)) OR (AB end stage liver disease))	9,026
**Query (expanded/display term):** ((((((((((MH "Palliative Care") OR ("Palliative Care")) OR (AB Palliative N3 Care)) OR (AB pallia*)) OR ("end of life care")) OR (AB end of life care)) OR (MH "Life Support Care+")) OR ("Support Care")) OR (MH "Symptom Burden")) OR ("Symptom Burden")	7,767
**Query (expanded/display term):** ((((((((((MH "Palliative Care") OR ("Palliative Care")) OR (AB Palliative N3 Care)) OR (AB pallia*)) OR ("end of life care")) OR (AB end of life care)) OR (MH "Life Support Care+")) OR ("Support Care")) OR (MH "Symptom Burden")) OR ("Symptom Burden") AND (((((TX (MH "Liver Cirrhosis+")) OR (XB ("liver cirrhosis"))) OR (AB liver N3 cirrhosis)) OR (AB hepatic N3 cirrhosis)) OR (AB end stage liver disease))	252
Limiters/Expanders/Last Run Via<br>Search run date (ISO): 2024-11-28-11T12:28:49.000Z<br>Filter(s):<br>Language: english<br>Age: all adult<br>Date (start date): 2014-01<br>Date (end date): 2024-11<br>Search mode: Find all my search terms<br>Expander(s): Apply equivalent<br>Number of databases: 1<br>Database(s): CINAHL with Full Text	117
**Search 2: updated 10.12.2025**	

**Query (expanded/display term):** ((((((((((MH "Palliative Care") OR ("Palliative Care")) OR (AB Palliative N3 Care)) OR (AB pallia*)) OR ("end of life care")) OR (AB end of life care)) OR (MH "Life Support Care+")) OR ("Support Care")) OR (MH "Symptom Burden")) OR ("Symptom Burden") AND (((((TX (MH "Liver Cirrhosis+")) OR (XB ("liver cirrhosis"))) OR (AB liver N3 cirrhosis)) OR (AB hepatic N3 cirrhosis)) OR (AB end stage liver disease))<br><br>Limiters/Expanders/Last Run Via<br>Search run date (ISO): 2025-12-10 T14:28:01.000Z<br>Filter(s):<br>Language: english<br>Age: all adult<br>Date (start date): 2024-11<br>Date (end date): 2025-12<br>Search mode: Find all my search terms<br>Expander(s): Apply equivalent<br>Number of databases: 1<br>Database(s): CINAHL with Full Text	10
**Search 3: updated 15.06.2026**	
**Query (expanded/display term):** ((((((((((MH "Palliative Care") OR ("Palliative Care")) OR (AB Palliative N3 Care)) OR (AB pallia*)) OR ("end of life care")) OR (AB end of life care)) OR (MH "Life Support Care+")) OR ("Support Care")) OR (MH "Symptom Burden")) OR ("Symptom Burden") AND (((((TX (MH "Liver Cirrhosis+")) OR (XB ("liver cirrhosis"))) OR (AB liver N3 cirrhosis)) OR (AB hepatic N3 cirrhosis)) OR (AB end stage liver disease))<br><br>Limiters/Expanders/Last Run Via<br>Search run date (ISO): 2026-06-15T09:10:10.000Z<br>Filter(s):<br>Language: english<br>Age: all adult<br>Date (start date): 2025-12<br>Date (end date): 2026-06<br>Search mode: Find all my search terms<br>Expander(s): Apply equivalent<br>Number of databases: 1<br>Database(s): CINAHL with Full Text	6
**Search 4: updated search including “advance care planning”, 01.01.2014–20.08.2026**	
**Query (expanded/display term):** ((((((((((MH "Palliative Care") OR ("Palliative Care")) OR (AB Palliative N3 Care)) OR (AB pallia*)) OR ("end of life care")) OR (AB “advance care planning”)) OR (AB end of life care)) OR (MH "Life Support Care+")) OR ("Support Care")) OR (MH "Symptom Burden")) OR ("Symptom Burden") AND (((((TX (MH "Liver Cirrhosis+")) OR (XB ("liver cirrhosis"))) OR (AB liver N3 cirrhosis)) OR (AB hepatic N3 cirrhosis)) OR (AB end stage liver disease))<br><br>Limiters/Expanders/Last Run Via<br>Search run date (ISO): 2026-08-20 T08:08:31.000Z<br>Filter(s):<br>Language: english<br>Age: all adult<br>Date (start date): 2014-01<br>Date (end date): 2026-08<br>Search mode: Find all my search terms<br>Expander(s): Apply equivalent<br>Number of databases: 1<br>Database(s): CINAHL with Full Text	135
