# Supplementary material for: Palliative Care in Patients with Liver Cirrhosis: A Scoping Review of Identification, Interventions, and Implementation Barriers and Facilitators
Source: Healthcare (Basel). 2026 Sep 1;14(17):2761. doi: 10.3390/healthcare14172761 (PMC13566022; doi:10.3390/healthcare14172761)
Supplement: Supplementary file 1 [file healthcare-14-02761-s001.zip › Table S2_data charting_proofread_BGJ.pdf]

Table S2: Data charting from the included studies

RQ1: How are patients identified for palliative care, and what interventions are provided for liver cirrhosis?

Studies N= 25

References	Year	Study design and aim	No. of participants	Cirrhosis etiology / inclusion	Characteristics of participant	Identification / Intervention	Outcome	Author(s) conclusion
Barnes et al.[35] (Australia)	2020	Retrospective single-centre cohort study.<br><br>Aim:<br>To examine EPCR, associated clinical factors, healthcare utilization, and survival.	N=74 patients	Alcoholic liver disease: 47%<br>NAFLD: 22%<br>Alcohol + HCV: 16%<br>HCV: 6%<br>Other: 9%	Male: 69%<br><br>Mean age: 59 y	**Identification:**<br>Inclusion: ≥2 admissions for decompensated liver disease within 6 months and ineligible for liver transplantation.<br><br>**Intervention:**<br>Early outpatient PC referral (≥30 days before death), including ACP/end-of-life discussions and symptom management.	EPCR occurred in 19% and was associated with increased outpatient PC involvement and ACP, and lower hospital resource use. No difference in survival.	EPCR may improve quality of care and reduce resource utilization in patients with ESLD. Further high-quality studies are needed
	2022	Retrospective multicentre cohort study.<br><br>Aim:<br>To evaluate the BPS for predicting 1-year mortality	N=276 patients	Alcohol-related liver disease: 64.9%<br>Other etiologies not specified	Male: 67.8%<br><br>Mean age: 61y	**Identification:**<br>BPS ≥3 indicating consideration of PC, based on Child–Pugh class C, ≥2 hospital admissions within 6 months, ongoing alcohol use, transplant ineligibility, and WHO performance status 3–4.	1-year mortality 37.3%<br><br>BPS sensitivity 54.9%, specificity 58%, PPV 43.4%, NPV 68.7%	BPS did not accurately predict 1-year mortality, highlighting challenges in prognostication in chronic liver disease.
Gaines et al. [37] (USA)	2025	12-week quality improvement project<br><br>Aim:<br>To identify unmet PC needs in non-transplantable liver disease patients by	N=16 patients	Non-transplantable liver disease / ESLD	Male: 31%<br><br>Mean age: 52 y	**Identification:**<br>NECPAL screening (including the SQ) combined with CLDQ<br><br>**Intervention:**<br>Nurse-led implementation of NECPAL screening	Approximately 60% screened positive on NECPAL indicating PC needs. CLDQ showed 80% with unmet symptoms (abdominal discomfort, worry, family impact)<br>Nurses and nurse practitioners reported the tools were easy to use and supported	Implementation of NECPAL identified substantial unmet PC needs in non-transplantable liver disease patients and supported integration of PC into routine nursing care to improve symptom management and end-of-life care.

		implementing the NECPAL and CLDQ tool into routine nursing assessment.					identification of symptom burden and unmet PC needs.	
Grønkjær et al. [55] (Denmark)	2026	Multicenter, mixed-method, non-randomized feasibility study.<br><br>Aim:<br>To evaluate feasibility of an early PC intervention for cirrhosis	N= 42 patients<br><br>N= 25 informal caregivers	Alcohol: 71%<br>MASLD/ MASH: 17%<br>Viral: 10%<br>Other: 7%.	Male: 30 (85%)<br><br>Mean age: 65 (71%)<br><br>Ascites: 63%<br><br>Child-Pugh A/B/C: 22/52/26<br><br>Mean MELD: 13	**Identification:**<br>Patients were identified using SPICT (≥1 general and ≥1 liver-specific indicator).<br><br>**Intervention:**<br>Two-day PC training for health care professionals. The intervention included structured PC conversations, ACP, PRO assessment, joint hepatologist–nurse initiation, nurse-led follow-up, and monthly specialist PC supervision.	Patient-reported outcomes stable during follow-up (PRO-pall, EQ-5D-5L and HADS). Caregiver burden decreased markedly (ZBI 25.1 -> 16.1).<br><br>Qualitative themes: improved understanding, emotional validation, future planning, reduced caregiver distress.	The intervention was feasible and acceptable to healthcare professionals, patients, and informal caregivers, supporting further evaluation in a larger study.
Homann et al. [38] (USA)	2025	Retrospective cohort study<br><br>Aim:<br>To assess the performance of SQ in patients diagnosed with end-stage liver disease	N= 378 patients	Alcohol use: 27.5%<br>Non-alcoholic steatohepatitis: 27.5%	Male: 60.1%<br><br>Mean age: 55.9 y<br><br>Mean MELD: 20	**Identification:**<br>Hepatologists used the SQ at hospital admission for ESLD patients: “Would you be surprised if this patient were to die in the next 12 months?” as a screening tool for mortality risk and potential PC needs.	Patients with a “No” response had lower 12-month survival (58.4% vs. 92.3%, p<0.001) and higher MELD scores. The SQ showed high sensitivity (93%) and NPV (92%), but low specificity (38%) and PPV (41%). Among readmitted patients, 80% of responses were consistent, and nearly half with repeated “No” responses died within one year.	SQ may be useful as a screening tool in ESLD within clinical assessment, but not as a standalone prognostic measure
	2016	Observational study with retrospective chart review and quality improvement design (PDSA cycles)<br><br>Aim:	N = 73 patients	Alcohol-related liver disease: 63%<br><br>Other etiologies were not specified in detail.	Male: 79,5%<br><br>Mean age: 54 y	**Identification:**<br>Prognostic screening tool based on five clinical criteria (Child–Pugh C, ≥2 admissions/6 months, ongoing alcohol use, transplant	A score ≥3 predicted 1-year mortality with 72.2% sensitivity, 83.8% specificity, and 81.3% PPV.	The prognostic screening tool identified patients at high risk of 1-year mortality and may support earlier integration of PC.

		To develop and validate a prognostic screening tool to identify hospitalized patients with decompensated cirrhosis at high risk of 1-year mortality and support implementation of supportive care.				ineligibility, and WHO PS 3–4), with a score ≥3 indicating high risk of 1-year mortality. of 1-year mortality		
Kearney et al. [40] (Australia)	2023	Prospective quality improvement study with retrospective control cohort.<br><br>Aim:<br>To evaluate PC delivery with the HepatoCare model versus SOC and whether modified SPICT-based referral criteria identified patients at high risk of death.	In total N=60 patients<br><br>N=30 SOC<br><br>N= 30 Hepato Care	**SOC**<br>Alcohol 9: (30%)<br>Non-alcohol-related: 21(70%)<br>Hep C-related: 14 (47%)<br>Ongoing alcohol consumption: 7 (23%)<br><br>**Hepatocare**<br>Alcohol: 12 (40%)<br>Non-alcohol-related: 18 (60%)<br>Hep C-related: 4 (13%)<br>Ongoing alcohol consumption: 5 (17%)	**SOC**<br>Male: 26 (87%)<br><br>Mean age: 63 y<br><br>Mean MELD: 14.6<br><br>**Hepatocare**<br>Male: 17 (57%)<br><br>Mean age: 64 y<br><br>Mean MELD: 14.4	**Identification:**<br>Modified SPICT-based referral criteria.<br><br>**Intervention:**<br>Integrated multidisciplinary HepatoCare clinic involving hepatology, PC, and pharmacy, including symptom management, ACP, and GOC.<br><br>Identification:<br>Modified SPICT-based referral criteria.	Modified SPICT-based referral criteria identified patients with advanced cirrhosis and high 12-month mortality and appeared more useful for patient identification than MELD or Child–Pugh scores alone.<br><br>**HepatoCare vs. SOC:**<br>ACP documentation 100% vs. 20%. PC referral 100% vs. 40%. Home/hospice death 80% vs. 30%.12-month mortality 33% vs. 67% Inpatient days 470 vs. 794. Deprescribing 81% vs. 10%.	The integrated HepatoCare model was associated with greater PC delivery, increased ACP uptake, and reduced healthcare utilization compared with SOC. SPICT-based referral criteria supported identification of high-risk patients and earlier PC integration. Further evaluation recommended.
Johnson et al.[42] (USA)	2023	Quantitative retrospective cohort study<br><br>Aim:<br>To examine hospital admissions before and after implementation of standardized criteria for PC consultation.	N=58 patients	Alcohol: 46.6%<br>Hep C: 24.1%<br>NASH/Cryptogenic: 19 %<br>Other: 10.3%<br>HCC: 5.3%	Male: 67,2%<br><br>Mean age: 52.2 y<br><br>Mean MELD:25.0	**Identification:**<br>Standardized referral criteria (≥2: Child–Pugh C, ≥2 admissions, ongoing alcohol use in alcohol-related cirrhosis, or transplant ineligibility).<br><br>**Intervention:**	Increased PC utilization (8%->33%) and hospice referrals (30.8% vs. 0%)<br>Decreased 30-day readmission (33.3% vs. 47.6%)<br>No hospital deaths among those seen by PC. Mortality not significantly different	Implementation of standardized PC consultation criteria for patients with ESLD increased PC utilization. Patients seen by PC had increased discharges with hospice services and decreased readmission rates.

						A Registered Nurse Case Manager - and social worker–facilitated PC consultation including symptom assessment, ACP, illness understanding, GOC, and optional social work/chaplain support.		
Kimbell et al.[43] (UK) *(Also included in RQ2)*	2018	A feasibility trial with a mixed-method evaluation<br><br>Aim:<br>To assess feasibility, acceptability and preliminary impact of a nurse-led supportive PC intervention for patients with advanced liver disease.	N=47 patients<br><br>N=27 family carers<br><br>N=13 case-linked professionals	ALD: 31<br>NAFLD: 5<br>Combination of causes: 1<br>Unknown: 1	Patients<br>Male: 31<br><br>Mean age: 60.5 y<br><br>Mean MELD: 16<br><br>Mean Child–Pugh:10	**Identification:** Clinical criteria (≥2 hospital admissions with complications of advanced liver disease, e.g. ascites, bleeding, encephalopath, infection, HCC)<br>**Intervention:** Six-month nurse-led supportive care intervention comprising home assessment, mainly monthly telephone follow-up, symptom and psychosocial support, care coordination, and anticipatory care planning. Nurses acted as case managers.	**Quantitative findings:** Patient outcomes were assessed using POS, EQ-5D-5L, HADS, and MQOL-B. Carer outcomes using POS-Carer and CQOL. Improvements in POS and EQ-5D-5L were reported. Updated anticipatory care plans increased from 29% to 85%, while no overall reduction in healthcare use was observed.	The intervention was feasible and acceptable to patients, carers, and professionals, supporting further evaluation in a randomized controlled trial.
Li et al. [57] (USA)	2026	Cross-sectional analysis of baseline data from a prospective observational study<br><br>Aim<br>To examine caregiver-reported prognostic communication	N=127 caregivers	Alcohol: 60%<br>MASLD: 27%<br>HBV/HCV: 13%<br>Other: 11%	Male: 37%<br><br>Mean age: 59 y<br><br>Spouses: 63%	None	Caregiver burden measured with ZBI-12 and anxiety/depression measured with HADS.<br><br>Frequent prognostic communication -> lower caregiver burden (ZBI-12 −4.82, p=0.01)<br><br>Adequate prognostic information -> lower caregiver burden (ZBI-12 −4.21, p=0.02)	Frequent, adequate, or high-quality prognostic communication was linked to lower caregiver burden or anxiety, but most caregivers had never discussed end-of-life care with the hepatologist. highlighting the need for improved

		and its association with psychological distress and caregiver burden.					High-quality prognostic communication -> lower caregiver anxiety (HADS-A −2.31, p=0.03)<br><br>No significant association with depression<br><br>85% of caregivers never discussed end-of-life care with hepatologist	prognostic communication
Oliveira et al. [44] (Portugal)	2025	Prospective cohort observational study<br><br>Aim:<br>To assess symptom burden in patients with ESLD, its association with disease severity, and the potential role of PC in symptom management.	In total N=44 patients<br><br>N=23 PC<br><br>N= 21 SOC	Alcohol-related cirrhosis: 72.7%	**PC**<br>Male: 82.6%<br><br>Mean age: 69.4 y<br><br>Mean MELD: 20.4<br><br>**SOC**<br>Male: 85.7%<br><br>Mean age: 59.9 y<br><br>Mean MELD: 16.8	**Intervention:** Comparison of PC referral vs standard care.<br><br>Symptom assessment over time using ESAS-r.<br><br>MELD-Na recorded for prognostic comparison but not used for referral or symptom assessment	High prevalence of symptoms at baseline (fatigue 77%, pain 43%, anorexia 43%). In the PC group reduction in pain intensity (86.7% -> 23.1%, p = 0.008) and fatigue severity (100% ->84.2%, p < 0.001) were reported.<br><br>No significant association between MELD-Na and symptom burden.	PC was associated with improvement in symptom management. Symptom burden was not correlated with MELD-Na, suggesting that clinical severity scores may not fully reflect PC needs. Routine symptom assessment may support earlier identification of needs
Oliveira et al. [45] (Portugal)	2025	Retrospective cohort study<br><br>Aim:<br>To determine predictors of home PC in ESLD patients, and to examine outcomes of home PC and factors associated with death at home.	In total N=75 patients<br><br>N= 33 Palliative homecare<br><br>N= 42 No homecare	ALD: 76%<br>Hep C: 13.3%<br>Hep B: 8%<br>Others: 2.7%	**Palliative homecare cohort:**<br>Male: 28 (84.8%)<br><br>Mean age: 72.5 y<br><br>Mean MELD: 17.45<br><br>Hepatocarcinoma: 90.9%<br><br>**No homecare cohort:**<br>Male: 30 (71.4%),<br><br>Mean age: 64.5 y<br><br>Mean MELD:19.79	**Intervention:** Specialist home-based PC with follow-up on symptoms, emergency episodes, and hospitalizations. Duration varied (median 75 days).	Emergency episodes reduced (2.61 -> 1.06, p=0.000).<br><br>Hospitalizations showed a non-significant decrease (0.94 -> 0.58, p=0.063).<br><br>Home deaths more frequent (66.7% vs. 33.3%, p=0.000)	Home PC was feasible in ESLD and was more often provided to older patients and those with hepatocarcinoma or complications. It was associated with fewer emergency visits and a higher likelihood of home death

					Hepatocarcinoma: 64.3%			
Orman et al. [46] (USA)	2022	Prospective cohort study.<br><br>Aim:<br>To examine associations between PC, patient-reported measures, and clinical outcomes in hospitalized patients with cirrhosis.	In total N=679 patients<br><br>N=74 received PC<br><br>N=605 received no PC	**PC**<br>ALD: 29.7%<br>Hep C: 3.5%<br>ALD/HCV 9.5%<br>NASH: 33.8%<br>Other: 13.5%<br><br>**No PC**<br>ALD 31.7%<br>Hep C: 13.4%<br>ALD/HCV: 7.6%<br>NASH: 32.4%<br>Other: 14.9%	**PC**<br>Male:46 (62.2)<br><br>Mean age: 58.8 y<br><br>Mean MELD: 25.2 (9.4)<br><br>**No PC**<br>Male: 338 (55.9)<br><br>Mean age: 57,3 y<br><br>Mean MELD: 20.0 (7.7)	**Intervention:**<br>Inpatient specialist PC consultation addressing symptom management, GOC, and ACP, referrals at discretion of primary team.<br><br>Follow-up:<br>90 days (readmissions, costs, mortality)	PC was received by 11% of patients, with consultation occurring at a median of hospital day 8.<br><br>Compared with no PC, the PC group had higher in-hospital mortality (22% vs. 4%), hospice discharge (31% vs. 1%), 90-day mortality (48.5% vs. 18.0%), and completion of advance directives (48.4% vs. 4.3%).<br><br>Fewer blood transfusions and endoscopies were reported in the PC group, while procedures/day did not differ significantly.	PC was infrequently used and occurred late, primarily among patients with more severe disease and functional impairment. PC was associated with lower healthcare utilization and greater completion of advance directives. Randomized trials were recommended.
Orman et al. [54] (USA)	2026	Single-centre pilot randomized controlled trial.<br><br>Aim:<br>To evaluate the feasibility of the Cirrhosis Medical Home (CMH), a collaborative care model for patients with decompensated cirrhosis, compared with usual care.	In total N=40 patients<br><br>N= 20 CMH<br><br>N= 20 usual care.<br><br>205 patients scree-ned<br><br>N=4 care-givers	Alcohol-related cirrhosis: 38%<br>MASLD: 48%<br>Hep C: 8%<br>Other: 8%	Male: 30%<br><br>Mean age: 58 y<br><br>MELD: 24<br><br>Child-Pugh C: 55%	**Identification:**<br>Patients hospitalized with decompensated cirrhosis (ascites or hepatic encephalopathy) and poor quality of life (SF-36 <40) were enrolled.<br><br>**Intervention:**<br>The CMH comprised nurse-coordinated multidisciplinary assessment of physical, cognitive, and psychological needs, individualized care planning, education, community resource linkage, and follow-up at	Primary outcomes: feasibility (enrolment, retention, completeness of data collection).<br><br>Secondary outcomes: quality of life (SF-36), frailty (SPPB, LFI), anxiety/depression (GAD-7, PHQ-9), cognition (PHES), healthcare utilization, mortality. Intention-to-treat analysis showed no significant differences in quality of life, readmissions, or mortality. Per-protocol analysis showed improvements in physical functioning, energy/fatigue, and physical component score at 3 months.	CMH was feasible to deliver, although recruitment, retention, and follow-up were challenging due to high mortality, transplantation, and loss to follow-up.<br>The model showed potential to improve patient-reported outcomes, but larger studies are needed to determine effectiveness.

						least every two weeks for six months.		
Patel et al. [56] (USA)	2020	Quality improvement pilot study.<br><br>Aim:<br>To improve advance directive (AD) completion and GCD documentation through a hepatologist-led ACP intervention and identify provider-reported barriers.	N= 57 patients<br><br>AD Completed<br>N= 14<br><br>AD Not Completed<br>N= 43	**AD completet**<br>Alcohol: 2 (9%)<br>HCV: 4 (29%)<br>HBV: 1 (100%)<br>NASH: 3 (38%)<br>Autoimmun: 1 (50%)<br>Cholestatic: 2 (66%)<br><br>**Ad Not Completed**<br>Alcohol: 20 (91%)<br>HCV: 10 (71%)<br>NASH: 5 (62%)<br>Autoimmun: 1 (50%)<br>Cholestatic: 1 (33%)	**AD completet**<br>Male: 16%<br><br>Mean age: 58.8 y<br><br>Mean MELD: 13.6<br><br>**Ad Not Completed**<br>Male: 84%<br><br>Mean age: 57.3 y<br><br>Mean MELD: 13.3	**Intervention:** Hepatologist-led outpatient ACP intervention including pre-visit identification of patients without an AD, ACP discussions, standardized EHR templates, staff education, and support for AD completion and follow-up.	AD completion increased from 8% to 31%, and GCD documentation increased from 0% to 51%. The most commonly reported provider barrier to ACP was needing more time during visits (38%). Other barriers included competing issues during the visit, late patient arrival, and patients needing more time to discuss ADs with family members.	Targeting provider- and system-specific barriers to ACP was associated with increased AD completion and GCD documentation. ACP discussions were considered feasible to incorporate into hepatology clinic visits.
Patel et al. [47] (USA)	2020	Retrospective chart review study<br><br>Aim:<br>To pilot the use of quality indicators to measure the quality of PC delivered to veterans with end-stage liver disease.	N= 62/167 patients screened positive	History of drug or alcohol use: 41 (66%)<br>Hep C: 37 (60%)<br>HCC: 10 (16%)	Male: 98%<br><br>Mean age: 61 y<br><br>MELD ≥20 or Child Pugh ≥12	No active intervention (observational chart review study)<br><br>Quality assessment via chart review using PC-ESLD quality indicators SPC/hospice access, ACP, documentation of treatment preferences, symptom management.	Overall, 68% of recommended PC measures were completed. 63% of patients who died had documentation of PC or hospice within 6 months before death.<br>ACP documented in about 50–55% of eligible patients.<br><br>Those receiving PC had more documented discussions and planning (67% vs. 37%).	Measuring PC quality for ESLD patients is feasible. Overall care is inadequate but appears better for those receiving specialist care
Patel et al. [58] (USA)	2026	Retrospective cohort study with Quality	N= 167,727 patients	Veterans with cirrhosis.	Male: 96%<br><br>Mean age: 69.1 y	**Identification:** Veterans Health Administration policy targeted	GCD documentation increased from 0.4% to 21.2% in the Quality Improvement period.	GCD documentation increased over time in the context of targeted QI initiatives. Future

		Improvement initiatives.<br><br>Aim:<br>To quantify the frequency and timing of GCDs, identify factors associated with GCD documentation, and examine changes in documentation in the context of targeted Quality Improvement initiatives.			Decompensated cirrhosis: 31%.	high-risk patients for whom a life-threatening clinical event within 1–2 years would not be unexpected.<br><br>**Intervention:**<br>GCDs addressing goals, values, illness understanding, and preferences for life-sustaining treatment, supported by standardized documentation, monitoring, staff education and training, and national guidance.on GCDs.	Overall, 29.8% had a documented GCD, increasing to 50.7% among patients with decompensated cirrhosis. GCDs were associated with liver-related hospitalization and specialist PC consultation.<br>Median time from first GCD to death was 37 days.	interventions should be conducted earlier and address illness understanding.
Reeves et al. [53] (Australia)	2026	Retrospective longitudinal cohort study.<br><br>Aim:<br>To determine the proportion of patients with decompensated cirrhosis who had PC needs, were referred to SPC, and the type of PC received.	N = 285 patients included<br><br>SPICT-positive: n = 179 (62%)	Alcohol use disorder: 55%	Male: 62%<br><br>Mean age: 56 y<br><br>Mean MELD: 14.6<br><br>Child-Pugh B/C: 71%	**Identification:**<br>Identification of PC needs using modified SPICT criteria followed by review of specialist PC involvement.	179 patients (62%) met modified SPICT criteria. Of these, 77 (43%) were referred to specialist PCat a median 50 days prior to death. Among referred patients, 71% received combined inpatient and outpatient PC, 13% inpatient care only, and 16% community-based care only. Median time to death among SPICT-positive patients was 373 days.	Modified SPICT criteria identified PC needs in a substantial proportion of patients, but more than half of those identified were not referred. Further research is needed to determine optimal referral timing and models of PC integration.

Shehada et al. [48] (USA)	2024	Retrospective cohort study<br><br>Aim:<br>To examine PC utilization and compare outcomes between patients with and without PC involvement. involvement.	In total N=576 patients<br><br>N=237 with PC (41.1%)<br><br>N=339 without PC (58.9%)	ESLD with complications such as variceal bleeding, ascites, SBP, HCC, hepatorenal syndrome, or hepatopulmonary syndrome. Liver transplant recipients excluded.	**PC group**<br>Male: 44,8%<br><br>Mean age: 64.1 y<br><br>**No-PC group**<br>Male: 42.6%<br><br>Mean age: 64.6 y	**Intervention:**<br>PC consultation addressing symptom management and GOC, including ACP, code-status changes, and healthcare proxy assignment. PC was typically initiated late in the disease course.	Hospital admissions decreased after PC (15.66 ->3.49 p<0.001).<br><br>Significant increase in GOC processes: More code-status changes in PC group (77.6% vs. 29.2%).<br><br>More health care proxy assignments (63.7% vs. 37.5%).<br>More symptom-focused procedures: Aspira catheters (5.9% vs. 0.9%), frequent paracentesis (30.8% vs. 16.8%).<br><br>Higher mortality in PC group (83.1% vs. 46.4%).	PC involvement was associated with fewer hospitalizations and greater attention to symptom management and GOC, despite often occurring late in the disease course. Earlier integration and further prospective evaluation were recommended.
Shinall et al. [51] (USA)	2019	Randomized controlled trial<br><br>Aim:<br>To evaluate the effect of specialist PC on hospital readmissions and other clinical and patient-reported outcomes in patients with ESLD	In total N=63 patients<br><br>N=31 Intervention<br><br>N=32 Control	NASH: 35%<br>Alcohol: 25%<br>Hep C: 16%<br>Hep B: 6%<br>Other: 18%	Male: 50%<br><br>Mean age: 58y<br><br>Mean MELD: 24	**Identification:**<br>Surprise Question (SQ); patients whose hepatologist answered “no” were identified as eligible for PC.<br>**Intervention:**<br>Inpatient specialist PC consultation addressing symptom management, GOC, and ACP, with post-discharge nurse telephone follow-up.	The trial was stopped early due to recruitment and implementation challenges. 63 of 293 eligible patients enrolled, and 44% of follow-up calls were completed.<br>Questionnaire data were insufficient for analysis.<br>No significant differences were found in unadjusted analyses. Adjusted analyses showed lower readmission risk (HR 0.36, p=0.017) and more days at home (OR 3.97, p=0.03) in the intervention group.	Despite substantial implementation challenges, the intervention was associated with longer time to first readmission and more days alive outside the hospital during the first six months.
Ufere et al. [49] (USA)	2022	A single-site pilot randomized controlled trial.<br><br>Aim:<br>To assess the feasibility, acceptability, and preliminary efficacy of an ACP video decision-support tool on knowledge and	Randomized in total N=50 patients<br><br>N=26 Video<br>N=24 Verbal	ALD: 64%<br>NASH/ Crypto-genic: 20%<br>Multifactorial/other: 16%<br>HCC: 4%	Male: 50%<br><br>Mean age: 60 y<br><br>Mean MELD: 16.5.	**Intervention:**<br>ACP video decision support tool presenting three GOC (life-prolonging care, limited medical care, comfort care). Control group received the same information verbally.	Preference for life-prolonging care decreased in both groups (video: 50% → 15%, p=0.029. Verbal: 50% → 29%, p=0.047).<br><br>Preferences for CPR (Video: 69% → 35%, p=0.01. Not significant in verbal group)<br><br>Intubation (Video: 62% → 23%, p=0.001. Not	The ACP video tool is feasible, acceptable, and shows promising early efficacy for improving knowledge and shaping end-of-life preferences in transplant-ineligible patients with advanced liver disease, warranting further evaluation in larger studies.

		end-of-life care preferences in transplant-ineligible patients with advanced liver disease.					significant in verbal group)	
Van Zyl et al. [50] (USA)	2023	Prospective pilot study with retrospective comparison group.<br><br>Aim:<br>To assess the feasibility and impact of PC integrated within a hepatology clinic (PCIH) on healthcare utilization, ACP, and hospice enrollment in patients with ESLD.	In total N=112 patients<br><br>N = 55 received PCIH<br><br>N = 57 received usual care	**PCIH**<br>Alcohol: 76.4%<br>Hep B: 1.8%<br>Hep C: 20%<br>Nonalcoholic liver disease: 14.6%<br><br>**Comparison group**<br>Alcohol: 80.7%<br>Hep B: 0<br>Hep C: 15.8%<br>Nonalcoholic liver disease: 10.5%	**PCIH**<br>Male: 69.1%<br><br>Mean age (SD)<br>PCIH: 52.5<br><br>Mean MELD: 24.8<br><br>**Comparison group**<br>Male: 71.9 %<br><br>Mean age: 52.6<br><br>Mean MELD: 24.6	**Intervention:**<br>PC integrated in a hepatology clinic including nurse practitioner and social worker providing symptom management, psychosocial assessment, care coordination, GCD, and ACP. Group education on ESLD and ACP provided.	PCIH patients had fewer hospital days (13 vs 19.7), more GOC discussions (87% vs 21%), more ACP documents (56% vs 7%), more psychosocial assessments (98% vs 35%), and higher hospice enrollment (26% vs 7%). No differences in mortality, ICU admission, or place of death.	Integrated PC within hepatology was feasible and acceptable and was associated with greater engagement in ACP and end-of-life care processes.
Verma et al. [59] (USA)	2026	Cluster randomized clinical trial (PAL-LIVER).<br><br>Aim:<br>To compare the effectiveness of PCdelivered by trained hepatologists with PC specialists in improving quality of life at 3 months.	In total N = 935 patients<br><br>N=419 hepatologist-led<br><br>N=516 SPC	**Specialist care:**<br>ALD: 39%<br>MASH: 24%<br>HBV/HCV: 27%<br>HCC: 31%<br><br>**Hepatologist-led palliative care:**<br>ALD: 39%<br>MASH: 32%<br>HCC: 24%	**Specialist care:**<br>Male: 76%<br><br>Mean age: 64 y<br><br>Mean MELD: 14.2<br><br>Child Pugh C: 18%<br><br>**Hepatologist-led PC**:<br>Male: 64%<br><br>Mean age: 62 y<br><br>Mean MELD: 15<br><br>Child-Pugh C: 33%	**Intervention:**<br>Four PC visits over 3 months delivered by either PC-trained hepatologists or SPC using a structured PC checklist. Intervention included symptom management, psychosocial support, and prognosis-related conversations.<br><br>Hepatologists received a 12-week liver-specific PC training programme.	Quality of life improved in both groups. Hepatologist-delivered PC was noninferior to specialist-delivered PC for improvement in FACT-Hep scores at 3 months. Symptom burden and depression improved in both groups without significant between-group differences. Patient satisfaction improved more in the hepatologist group. Mortality was similar between groups.	PC delivered by trained hepatologists was comparable to specialist-delivered PC in improving quality of life and was associated with greater improvement in patient satisfaction. The findings support hepatologist-delivered primary PC as an effective model for patients with ESLD.
Ward et al. [41] (UK)	2025	Single-centre quality improvement	In total N= 77 patients	Alcohol-related: 76%	PDSA 2<br>Male: 76%	**Identification:**<br>Hospitalized patients with end-stage liver	PDSA1:<br>Significant reduction in patients receiving no PC measures (67% → 26%),	SPICT can improve access to PC, particularly when linked to direct action

		project (two PDSA cycles)<br><br>Aim:<br>To evaluate and enhance access to PC for inpatients with end-stage liver disease and unmet PC needs over a 6-month period using a novel SPICT-based intervention	N=27 PDSA cycle 1<br><br>N= 50 PDSA cycle 2	NAFLD: 24%	Mean age: 56 y	disease meeting SPICT criteria<br><br>PDSA 1:<br>SPICT used to trigger direct specialist PC referral<br><br>PDSA 2:<br>SPICT used to trigger multidisciplinary meetings (MDM) between hepatology and PC.	increase in SPC input (32% → 52%) and community PC referral (13% → 39%).<br><br>PDSA2:<br>No significant improvement. Only 42% discussed in MDM, low ACP and community referral rates	rather than complex MDT processes
Warmels et al. [52] (Canada)<br>*(Also included in RQ2)*	2025	Convergent parallel mixed methods process evaluation.<br><br>**Aim:**<br>To evaluate a novel outpatient PC clinic for patients with ESLD	Chart review: N=46 patients<br><br>Inter-views: N= 8 healthcare providers (HCP)<br><br>N=13 patients /caregiver	Alcohol-associated liver disease: 23.9%<br>Viral hepatitis: 15.2%.<br>All patients had decompensated cirrhosis.	Male: 63%<br><br>Mean age: 73 y	**Intervention:**<br>Integrated outpatient hepatology–PC clinic led by specialist PC physicians, providing symptom management, GOC discussions, future planning, medication review, and care coordination.<br>The clinic evolved from monthly consultations to a shared-care model with weekly availability and telephone support.	**Quantitative outcomes:**<br>Emergency department visits and hospitalizations decreased after consultation. 15/24 (62.5%) deceased patients died outside hospital.	The outpatient PC clinic provided collaborative care and was associated with reduced acute healthcare utilization and more deaths outside hospital, supporting the model as a promising approach to PC in ESLD.

RQ2: What are the key facilitators and barriers to implementing palliative care interventions for patients with liver cirrhosis from the perspectives of patients, informal caregivers and healthcare professionals?

Studies N= 16

References	Year	Study design and aim	No. of partici-pants	Cirrhosis etiology / inclusion	Characteristics of participant	Barriers and facilitators to PC implementation	Author(s) conclusion
Barber et al. [72] (Canada)	2026	Qualitative cross-sectional study using Cognitive Task Analysis. Semi-structured interviews<br><br>Aim:<br>To explore family physicians' approaches to symptom management in cirrhosis and the integration of PC principles in primary care.	N=6 family physicians	Not applicable	Male: 2<br><br>Age range: 30-69 y	**Contextual findings:** Family physicians described cirrhosis care as reactive and case-by-case.<br>**Barriers:** Lack of structured processes. Unclear roles and weak connections with specialist care. Limited connections with specialist care. Disease complexity, comorbidities, socioeconomic challenges, and limited supportive resources further complicated symptom management. GOC and ACP discussions were inconsistent and often dependent on patient readiness or clinical deterioration. Need for additional information and support.<br><br>**Facilitators:** Physicians considered GOC and ACP discussions part of their professional role.	Family physicians lacked clear structures and tools for cirrhosis symptom management, leading to unclear roles, reactive care, and limited integration of palliative principles.
Carbonneau et al. [60] (Canada)	2018	Qualitative interview study<br><br>Aim:<br>To explore patients' experiences and perceptions with ACP/GCD.	N=17 patients	AIH : 1<br>Non-alcoholic: 4<br>Alcohol : 8<br>Hep C : 2<br>Alcohol/Hep C: 1<br>Cryptogenic: 1	Male:14<br><br>Mean age: 56.8 y<br><br>Mean MELD: 12.1	**Barriers:** Lack of understanding about disease trajectories and ACP processes. Recall of previous ACP/GCD discussions was often limited when discussions had occurred during acute illness. Discussing end-of-life issues with family was challenging, and some patients associated ACP with deterioration or loss of hope.<br><br>**Facilitators:** Patients preferred discussions outside hospital and not during acute illness. Most preferred realistic, direct information.	The findings support the need for early ACP in the stable outpatient setting. Cirrhosis and primary care clinicians may play a key role in facilitating these discussions in their clinics, involving alternate decision makers, educating patients using high quality resources, and understanding their preferences.
Donlan et al. [61] (USA)	2021	Qualitative semi-structured interviews with patients with ESLD (transplant-eligible and ineligible) and their caregivers<br><br>Aim<br>To explore perceptions of PC.	N= 15 patients<br>(8 transplant-eligleble / 7 transplant-ineligible)<br><br>N=14 caregivers	Alcohol: 7<br>NASH: 5<br>Other: 3	Male: 5<br><br>Mean age: 60 y<br><br>Mean MELD: 14	**Barriers:** PC was often perceived as end-of-life care and as stopping active treatment. Participants had limited knowledge of PC and often associated it with hospice care.<br><br>**Facilitators:** Education (receiving a standardized definition of PC) increased awareness that PC can focus on quality of life and be provided alongside curative therapy.	Education is needed to clarify PC's role and enable earlier integration in ESLD care.

			(12 were patient/care giver dyads).			No differences were identified between patients and caregivers.	
Donlan et al. [62] (USA)	2025	Cross-sectional analysis of an ambulatory cohort.<br><br>Aim:<br>To examine patients' experiences of prognostic communication and its associations with symptom burden, psychological distress, and HRQOL.	N=218 patients	Alcohol: 127 (58.3)<br>MASLD: 50 (22.9)<br>HBV or HCV: 29 (13.3)<br>Others: 12 (5.5)	Male: 118<br><br>Mean age: 60 y<br><br>Mean MELD: 16	**Contextual findings**:<br>Prognostic communication was considered very/extremely important by 93%, but only 41% reported frequent prognostic conversations, 81% had never discussed their end-of-life care wishes with their hepatologist, and 37% wished they had more prognostic information. Among patients who had discussed their prognosis, 78–88% considered prognostic information very/extremely helpful for treatment decisions, preparing for the future, maintaining hope, and coping with their disease.<br><br>**Barriers:** Infrequent prognostic conversations, insufficient prognostic information, and limited end-of-life care discussions.<br><br>**Facilitators:** Patients valued prognostic communication and reported that knowing their prognosis was helpful for treatment decisions, preparing for the future, maintaining hope, and coping with their disease.	Patients valued prognostic communication but reported infrequent discussions.<br>Inadequate communication was associated with greater symptom burden and psychological distress and lower HRQOL, highlighting opportunities to improve prognostic communication in ESLD care.
Hoppmann et al. [66] (USA)	2025	Embedded qualitative evaluation within the 19-site PAL-LIVER cluster randomized trial using semi-structured interviews and thematic analysis.<br><br>Aim:<br>To explore hepatologist and PC clinicians' experiences delivering PC to patients with advanced liver disease to guide future implementation.	In total N=32<br><br>N=17 hepatologists<br><br>N= 15 PC-specialists	Not applicable	Clinicians<br>Male: 34,4%<br><br>Mean age: 47.3 y<br><br>Physicians: 25%<br><br>Nurse practitioners: 43.7%<br><br>≤10 years practice	**Barriers:** Integrating PC requires time. PC specialists observed persistent misperceptions about PC among patients, families, and other clinicians.<br><br>**Facilitators:** Cross-disciplinary education. Strong hepatology–PC collaborations facilitated integration, early referral, and coordinated care.	Integration of PC into routine ALD care requires cross-disciplinary education, schedule adjustments to allow hepatologists to address PC needs, and discernment of which patients need primary vs specialty PC. Misperceptions of PC can be mitigated through strong hepatology-PC collaborations.
Hudson et al. [64] (UK)	2018	Qualitative study – semi-structured interviews using thematic analysis<br><br>Aim:	N=17 Patients<br><br>N=5 Caregivers	Alcohol: 8<br>NASH: 4	**Patients**<br>Male: 9<br><br>Mean age: 59.6 y<br><br>**Caregivers**	**Barriers:** Patients often lacked full understanding of their diagnosis and had limited opportunities for future planning. Attitudes toward ACP varied. Some associated it with "giving up." Caregivers reported inadequate symptom management, limited	Existing healthcare services are often not adapted to meet the palliative needs of patients with advanced liver disease.

		To explore supportive and PC needs among patients and family members			Male: 2<br><br>Mean age: 57.4 y	information about disease progression, and insufficient support after the patient's death were reported.<br><br>**Facilitators:** Some patients viewed ACP as helpful. Caregivers were generally more positive about ACP and felt it could have supported better preparation.	
Kimbell et al. [65] (UK)	2015	Qualitative, semistructured interviews and grounded theory techniques.<br><br>Aim:<br>To explore experiences, needs, and priorities of patients, caregivers, and HCPs towards the end of life to inform timely supportive and PC.	N= 15 Patients<br><br>N= 11 Lay carers<br><br>N= 11 HCPs (General practitioners: 8 Consultant hepatologist: 1 Hepatologist, hospital-based alcohol liaison nurse:1 Community PC nurse:1)	ALD: 6<br>ALD/HCV: 2<br>ALD/NAFLD: 1<br>NAFLD: 3<br>HCC/NAFLD: 1<br>AIH: 1<br>Cryptogenic: 1	**Patients**<br>Male: 6<br><br>Mean age: 58.8 y<br><br>Mean MELD: 16.3	**Patients**<br>**Barriers:**<br>Limited understanding of their disease, found explanations too medicalised, and were poorly informed about progression, symptoms, and support.<br>Uncertainty related to symptoms, progression, communication, and care dominated their experiences and made coping and planning difficult.<br><br>**Lay carers**<br>**Barriers:** Uncertainty related to the unpredictable illness, limited information, and complexities of care. This uncertainty influenced coping and made it difficult to identify support needs and engage in care planning.<br><br>**HCP**<br>**Barriers:** Uncertainty related to prognosis, timing of palliative discussions, and their role in care. Lack of awareness of support services.	Living, dying, and caring with advanced liver disease is dominated by pervasive uncertainty, which shapes experiences and actions.<br>Acknowledging this uncertainty can support coping, care planning, and timely provision of supportive and palliative care
Kimbell et al.[43] (UK)<br>*(Also included in RQ1)*	2018	A feasibility trial with a mixed-method evaluation<br><br>Aim:<br>To assess feasibility, acceptability and preliminary impact of a nurse-led supportive PC intervention for patients with advanced liver disease.	N=47 patients<br><br>N=27 family carers<br><br>N=13 case-linked professionals	ALD: 31<br>NAFLD: 5<br>Combination of causes: 1<br>Unknown: 1	Patients<br>Male: 31<br><br>Mean age: 60.5 y<br><br>Mean MELD: 16<br><br>Mean Child–Pugh:10	**Patients and carers**<br>**Barriers:** Poor understanding of the illness and its likely course hampered anticipatory care planning. Some patients and carers would have preferred nurse support earlier and more frequent face-to-face contact.<br>**Facilitators:** Patients and carers valued the support, including home visits and telephone follow-up.<br><br>**Professionals**<br>**Facilitators:** Professionals valued care coordination, timely advice, and support with decisions about hospital admission.	The intervention was feasible and acceptable to patients, carers, and professionals, supporting further evaluation in a randomized controlled trial.
Low et al. [67] (UK)	2016	On-line survey with closed and open responses<br><br>Aim:	N=514 HCPs<br><br>(6181 contacts	Not applicable	Not applicable	**Barriers:** Referrals to SPC were rare and mostly near end of life, reflecting cirrhosis' unpredictability, limited knowledge, and poor communication. Patients and families often lacked	All respondents acknowledged the role of SPC in caring for those dying with cirrhosis and need for

		To explore SPC referral practices, HCP training needs, approaches to improving end-of-life care in cirrhosis, and priorities for future research.	received the survey)<br><br>N=195 Liver professionals<br><br>N= 273 SPC<br><br>N= 46 General practice professionals			understanding of disease severity, while clinicians felt uncertain about discussing it.<br><br>**Facilitators:** Better information could support open conversations on prognosis and care choices.<br>Training is needed in liver-specific symptom control, pharmacology, and palliative communication. Improvements include earlier SPC involvement, stronger hospital–community collaboration, and clear EoL pathways.	further training to improve confidence and enable joint working between SPC, general practice and liver teams. The low response rate limits generalizability and warrants further validation.
Low et al. [71] (UK)	2017	Mixed methods, including a retrospective case note review, qualitative focus groups and individual interviews<br><br>Aim:<br>To examine care in the last year of life for patients with advanced cirrhosis and identify barriers and opportunities for improving PC within hepatology	N= 30 patients<br><br>N= 22 liver health professionals (13 focus group. 9 individual interviews)	ALD: 11<br>Hep C/ALD: 3<br>HCC/Hep C/ ALD:1<br>HCC/ HepB/Hep C/ALD: 1<br>Hep C: 4<br>Hep C/ HCC: 3<br>NASH 2<br>NASH/ HCC: 1<br>Other: 2	**Patients**<br>Male: 20<br><br>Patients Mean age : 58 y<br><br>Mean MELD: 23	**Care context:** Patients experienced frequent hospitalizations, late prognosis discussions, limited involvement in care planning, and late SPC referral (median 5 days before death).<br><br>**Barriers:** Prognosis and future-care discussions were rarely initiated by clinicians. Quality-of-life and PC topics were seldom addressed unless patients raised them. The unpredictable disease course made timing of conversations difficult. Patients often viewed the specialist centre as a "last chance," influencing expectations, and PC was perceived by some as equivalent to "giving up." Limited continuity of care due to rotating staff and fragmented information flow. Clinicians reported low confidence in discussing prognosis and PC.<br><br>**Facilitators:** Suggested improvements included integrated clinics, multidisciplinary meetings, and clearer tools/guidelines.	Despite unpredictable disease trajectories, patients experienced frequent hospitalizations and increasing symptom burden near the end of life. Earlier identification of irreversible deterioration and coordinated hepatology–PC may improve care.
Sprange et al. [68] (USA)	2020	Survey study among outpatients with cirrhosis using structured questionnaires and medical record data.<br><br>Aim:<br>To assess patients` familiarity with and readiness to engage in ACP and	N= 97	Alcoholic hepatitis: 40%<br>NAFLD: 28%<br>Hep B/C: 16%<br>Other causes (autoimmune hepatitis, primary biliary	Male: 57%<br><br>Mean age: 62 y<br><br>Mean Child-Pugh: 6.19<br><br>Mean MELD: 11.62	**Care context:** 43% had heard of ACP, 23.8% had a good understanding, and 18% recalled participating in an ACP conversation. Most discussions occurred with HCPs, primarily hepatologists and hospitalists.<br><br>**Barriers:**<br>None explicitly identified.<br><br>**Facilitators:** Patients rated key qualities in ACP discussions highly, including communication skills, empathy, information provision, medical expertise, and honest	ACP completion remains low among cirrhosis patients. Patients prefer early, outpatient, practitioner-led ACP discussions. More proactive integration of ACP into standard cirrhosis care is needed.

		completion of personal directives and GCD.		cirrhosis, etc.): 16%		prognosis. The majority preferred ACP conversations early in the disease course and in an outpatient setting.	
Standing et al. [69] (UK)	2017	Qualitative interview study using thematic analysis.<br><br>Aim:<br>To explore GPs' experiences and perceptions of how primary care can enhance end-of-life care for patients with liver disease	N=25 GPs	Not applicable	Male:12<br><br>Years of experience as GP<br><5years N= 5<br>5–10 years N=10<br>16–25years N=9<br>>25years N=1<br><br>Specialist hepatology/ gastroenterology experience or training N=4	**Barriers:**<br>Limited involvement and experience in liver disease due to predominantly hospital-led care. Uncertainty about prognosis and unclear communication from specialists hindered timely PC planning.<br><br>**Facilitators:**<br>GPs were willing to take a greater role in end-of-life and home-based care. Improved collaboration, clear care pathways, and access to specialist support (e.g., nurses and symptom management) were identified as important for PC provision.	End-of-life care for patients with liver disease requires greater attention due to complex symptoms and substantial social challenges, highlighting the need to improve care provision.
Malik et al. [63] (USA)	2025	Qualitative phenomenological study using semi-structured Using thematic analysis<br><br>Aim:<br>To explore hospitalized patients' experiences and perspectives on communication, PC, ACP, healthcare experiences, and the psychosocial impact of ESLD.	N=15 patients	Not explicitly reported (patients with ESLD)	Male: 8<br><br>Mean age: 61.4 y<br><br>Child–Pugh C	**Contextual findings**:<br>ESLD caused significant emotional distress, fear, and loss of autonomy.<br>**Barriers:**<br>Variation in communication and information. Limited initial understanding of PC. Varied awareness of ACP, and insufficient coordination among care providers.<br>**Facilitators:** Clear and empathetic communication. Family involvement in care and decision-making. Improved understanding of PC following explanation, and coordinated care among healthcare providers.	Patients with ESLD had unmet emotional, informational, and psychosocial needs. Earlier integration of PC, clear communication, timely ACP, family involvement, and coordinated care may support more holistic, patient-centered care.
Saravanan et al. [73](USA)	2026	Cross-sectional survey study.<br><br>Aim:<br>To examine multidisciplinary clinician perspectives on PC integration in ESLD, including timing, terminology, symptom management,	N=55 HCP	Not applicable	Healthcare professionals from transplant hepatology, transplant surgery, gastroenterology, general internal medicine, and	**Care context:**<br>Clinicians providing direct care to adults with ESLD, defined as cirrhosis with ≥1 complication (ascites, hepatic encephalopathy, variceal hemorrhage, or jaundice).<br><br>**Barriers:** Differences between specialties regarding timing of PC integration. Unrealistic caregiver expectations about prognosis or survival. Perceived lack of PC expertise in managing ESLD symptoms. Differences in confidence	PC integration in advanced ESLD was challenged by differences in professional roles, competencies, and perceptions of PC. Improved interdisciplinary collaboration and education may help promote earlier and more consistent integration of PC.

		perceived barriers, and role clarity.			palliative care.	in symptom management and assessment of goals, and differences regarding responsibility for PC delivery.<br><br>89% of clinicians agreed that PC is critical for transplant-ineligible patients.	
Ufere et al. [70] (USA)	2019	A cross-sectional survey of hepatologists' and gastroenterologists' who provide care to patients with ESLD<br><br>Aim:<br>To examine physicians' attitudes toward PC in ESLD and whether attitudes differed by liver transplant candidacy.	N=396 (32% response rate)	Not applicable	Male: 73%<br><br>Transplant hepatologists: 60%<br><br>General hepatologists: 30%<br><br>None had formal PC training.	**Contextual findings:** Most physicians supported access to PC for patients with ESLD (95%) and trusted PC clinicians (86%), but few frequently collaborated with inpatient (32%) or outpatient (11%) PC services. Only 48% would use PC for patients on the liver transplant waiting list.<br><br>**Barriers:** Concerns about patients' negative perceptions of PC, including fear, anxiety, association with no further disease-directed treatment, and discussions about dying. Physicians also held more negative views of PC for liver transplant candidates.<br>**Facilitators:** Strong physician support for access to PC. Trust in PC clinicians, and recognition that PC may be beneficial earlier in the disease course.	Most hepatologists and gastroenterologists support PC for ESLD patients but rarely collaborate with PC and are concerned about patient perceptions. Interventions are needed to improve collaboration and address misperceptions.
Warmels et al. [52] (Canada) *(Also included in RQ1)*	2025	Convergent parallel mixed methods process evaluation.<br><br>**Aim:**<br>To evaluate a novel outpatient PC clinic for patients with ESLD	Chart review: N=46 patients<br><br>Interviews: N= 8 HCP<br><br>N=13 patients/caregiver	Alcohol-associated liver disease: 23.9%<br>Viral hepatitis: 15.2%.<br>All patients had decompensated cirrhosis.	Male: 63%<br><br>Mean age: 73 y	**Patients/caregivers**<br>**Barriers:**<br>Burden of travel associated with in-person appointments and difficulty distinguishing the PC team from other care teams.<br>**Facilitators:**<br>PC physicians were reported as accessible, compassionate, and supportive.<br><br>**HCPs**<br>**Barriers:** Concerns that future clinic growth could be limited by physical clinic space and physician time.<br>**Facilitators:** Hepatologists reported high collaboration and effective communication with the PC team.	The outpatient PC clinic provided collaborative care and was associated with reduced acute healthcare utilization and more deaths outside hospital, supporting the model as a promising approach to PC in ESLD.

Abbreviations:

ACP = advance care planning

AD = advance directive

AIH = autoimmune hepatitis

ALD = alcohol-associated liver disease

BPS = Bristol Prognostic Score

CLDQ = Chronic Liver Disease Questionnaire

CQOL = carer quality of life

EQ-5D-5L = EuroQol 5-dimension 5-level

EPCR = Early Palliative Care referral

ESAS-r = Edmonton Symptom Assessment System revised

ESLD = End-stage liver disease

FACT-hep = Functional Assessment of Cancer Therapy-Hepatobiliary

GAD-7 = General anxiety disorder

GCD = Goals of Care Designation

GOC = Goals Of Care discussions

GP = General practitioners

HADS = Hospital Anxiety and Depression Scale

HCC = Hepatocellular carcinoma

Hep B/HBV = Hepatitis B virus

Hep C /HCV = Hepatitis C virus

HCP = Healthcare Professional

LFI = Liver frailty index

HRQOL = Health-related quality of life

MASLD = Metabolic dysfunction-associated steatotic liver disease

MELD = Model for End-Stage Liver Disease

MQOL-B = McGill Quality of Life Questionnaire - Expanded/B version

NAFLD = Non-alcoholic fatty liver disease

NASH = Non-alcoholic steatohepatitis

NECPAL = Necesidades Paliativas / Palliative Needs

NPV = Negative Predictive Value

PC = Palliative Care

PD = Personal directive

PHES = Psychometric hepatic encephalopathy score

PHQ-9 = Patient Health Questionnaire

POS = Palliative care Outcome Scale

POS-Carer = PCOutcome Scale – Carer version

PTPQ = Prognosis and Treatment Perceptions Questionnaire

PPV = Positive Predictive Value

RNCM = Registered Nurse Case Manager

SF-LDQOL = Short Form Liver Disease Quality of Life

SOC = Standard of Care

SPC = Specialist Palliative Care

SPICT = Supportive and Palliative Care Indicators Tool

SPPB = Short Physical Performance Battery

SQ = Surprise Question

ZBI-12 = Zarit Burden Interview-12
